# Factors influencing cranial variation between prehistoric Japanese forager populations

**DOI:** 10.1007/s12520-023-01901-6

**Published:** 2023-12-13

**Authors:** L. T. Buck, L. P. Menéndez, I. De Groote, B. R. Hassett, H. Matsumura, J. T. Stock

**Affiliations:** 1 Research Centre for Evolutionary Anthropology and Palaeoecology, Liverpool John Moores University, Byrom Street, Liverpool, L3 3AF UK; 2 Department of Anthropology of the Americas, University of Bonn, Oxfordstrasse 15, 53111 Bonn, Germany; 3 Department of Evolutionary Biology, University of Vienna, Djerassiplatz 1, 1030 Vienna, Austria; 4 Department of Archaeology, Ghent University, Sint-Pietersnieuwstraat 35, 9000 Ghent, Belgium; 5 University of Central Lancashire, Fylde Rd, Preston, PR1 2HE Lancashire UK; 6 Natural History Museum London, Cromwell Road, London, SW7 5BD UK; 7 School of Health Sciences, Sapporo Medical University, S1W17, Sapporo, 0608556 Japan; 8 Department of Anthropology, Western University, London, ON N6A 3K7 Canada

**Keywords:** Diet, Morphological variation, Jomon, Foragers

## Abstract

Understanding the factors shaping human crania has long been a goal of biological anthropology, and climate, diet, and population history are three of the most well-established influences. The effects of these factors are, however, rarely compared within a single, variable population, limiting interpretations of their relative contribution to craniofacial form. Jomon prehistoric foragers inhabited Japan throughout its climatic and ecological range and developed correspondingly varied modes of subsistence. We have previously demonstrated that a large sample of Jomon crania showed no clear climatic pattern; here, we examine variation in Jomon crania in more detail to determine if dietary factors and/or population history influence human intrapopulation variation at this scale. Based on well-established archaeological differences, we divide the Jomon into dietary groups and use geometric morphometric methods to analyse relationships between cranial shape, diet, and population history. We find evidence for diet-related influences on the shape of the neurocranium, particularly in the temporalis region. These shape differences may be interpreted in the context of regional variation in the biomechanical requirements of different diets. More experimental biomechanical and nutritional evidence is needed, however, to move suggested links between dietary content and cranial shape from plausible to well-supported. In contrast with the global scale of human variation, where neutral processes are the strongest influence on cranial shape, we find no pattern of population history amongst individuals from these Jomon sites. The determinants of cranial morphology are complex and the effect of diet is likely mediated by factors including sex, social factors, and chronology. Our results underline the subtlety of the effects of dietary variation beyond the forager/farmer dichotomy on cranial morphology and contribute to our understanding of the complexity of selective pressures shaping human phenotypes on different geographic scales.

**Supplementary Information:**

The online version contains supplementary material available at 10.1007/s12520-023-01901-6.

## Introduction

To understand human evolution, we must clarify which stressors were important drivers of adaptation during the dispersals and colonisations that have been so fundamental to our species (Wells and Stock 2007; Antón and Kuzawa 2017; Roberts and Stewart 2018; Buck et al. 2019). Understanding the interplay of factors resulting in human phenotypic variation is a long-standing challenge, and climate, diet, and population history are all well-established influences on cranial morphology. The effects of these factors are rarely compared within a single, variable population, however, complicating interpretations of their relative contribution. Here, we seek to explore the effects of climate, diet, and population history on a single sample from a geographically and climatically diverse culture with considerable dietary variation: prehistoric Jomon foragers from Japan.

Climate is a potential source of environmental stress during range expansion for any species, and we have previously shown that non-human primates (Japanese macaques, *Macaca fuscata*) share human ecogeographic patterns of cranial adaptation (Buck et al. 2018). Japanese macaques from colder, drier climates differ from their counterparts in warmer, more humid regions by having larger body size, shorter limbs, more rounded neurocrania, broader faces and taller, and narrower noses. These ecogeographic patterns resemble those seen in humans suggesting that, at a species-wide level, skeletal climatic adaptation in humans is conserved and resembles that of other primates (Buck et al. 2018). In contrast to a geographically comparable sample of Japanese macaques, however, we found no relationship between cranial shape and climate within a human sample of Jomon foragers who would have experienced equivalent climatic stress (Buck et al. 2019). This suggests that, in relation to climate, higher levels of stress are required to trigger equivalent phenotypic adaptation in humans compared to non-human primates. This difference in adaptive strategy likely results at least in part from more extensive human behavioural buffering against climate (Buck et al. 2019). When climatic stresses are largely mitigated by human behaviour, resulting in the lessening or absence of climatic selection on the skeleton, differences in behaviour itself may play a greater role in shaping morphology. In the absence of a clear climatic pattern in Jomon cranial shape, we seek here to clarify what other covariates may have affected variation amongst the Jomon at an interregional geographic scale by testing the influences of diet and population history.

### Climatic influence on Jomon cranial morphology

The Jomon people were foragers who inhabited the Japanese Archipelago from ca. 14,500 BP to ca. 300 BP (Habu 2004). There is a substantial body of research investigating Jomon cranial shape, but opinions differ as to the structure of variation between sites and as to the potential role of climate. Our own work (Buck et al. 2019) notwithstanding, widescale geographic comparisons of Jomon cranial morphology with an explicitly climatic framework are lacking, despite considerable research into the effects of climate on Jomon postcranial variation. For example, postcranial analyses have shown greater body size and estimated mass are significantly correlated with latitude, in keeping with Bergmann’s Rule (Temple and Matsumura 2011; Fukase et al. 2012). In contrast to our previous analysis of cranial morphology (Buck et al. 2019), some authors have found results consistent with an ecogeographic component to differences in cranial morphology. Matsumura et al. (2001) reported larger vaults and orbits in Jomon from Hokkaido compared to those from further south, morphology fitting with cold-adaptation seen in other populations (Beals 1972; Pearce and Dunbar 2012). Other researchers contend that high levels of variation within as well as between regions obscure any clear inter-regional pattern (Kondo 1994). The potential influence of gene flow from outside the Japanese Archipelago, complicating regional patterns of variation, has been suggested (Mizoguchi and Dodo 2001; Hanihara and Ishida 2009) but more recent analyses with additional ancient genomes downplay this possibility (Kanzawa-Kiriyama et al. 2013; Adachi et al. 2021; Mizuno et al. 2023). Part of the disagreement regarding Jomon regional variation may be caused by various authors using different samples and measurements and comparing them at different geographic scales. Thus, methods, sample composition, and comparison choice may have affected the results. Here, we use the same Jomon individuals that we previously analysed for climatic influence (Buck et al. 2019) to investigate the influences of both diet and population history, thereby avoiding issues associated with sampling bias.

### Dietary influence on cranial morphology

Our first research question is: are there significant differences in Jomon cranial shape between dietary groups? Dietary influences on cranial shape have been documented at an intrapopulation to global scale and the division most frequently investigated is that between agricultural and forager diets. Morphological differences between farmers and foragers are well documented across different chronological periods and geographical regions (Paschetta et al. 2010; Katz et al. 2017; Menéndez and Buck 2022). The effects of diet, however, go beyond this dichotomy, with more subtle variations in subsistence practises also associated with demonstrable morphological differentiation (Menéndez et al. 2014; Katz et al. 2017; Menéndez and Buck 2022). For example, Katz et al. (2017) found that within pre-industrial agricultural subsistence practises, dairying had a particular influence on cranial morphology and measurable differences have also been found in the morphology of those following meat- or protein-based, versus plant- or carbohydrate-based diets (Menéndez et al. 2014; Noback and Harvati 2015). Variation in dietary biomechanical properties and the resulting differences in masticatory strain are held to be key factors in diet-related skull shape variation worldwide, and this relationship can be inferred from morphological, archaeological, or ethnographic evidence (Noback and Harvati 2015; Katz et al. 2017). The nutritional composition of diet, particularly the proportion of protein, has also been shown to play a role in cranial size and shape variation (Sardi et al. 2006; Menéndez et al. 2014). Thus, differences in cranial morphology between dietary groups inconsistent with differences in biomechanical properties may still relate to diet.

The Jomon are generally classified as broad-spectrum foragers, yet the ecological range of the Japanese Archipelago led to much variation in regional diet with differing dependence on marine, freshwater aquatic, and terrestrial proteins, and on plant foods, depending on their availability (Akazawa 1986a, 1986b, 1988, 1999; Akazawa and Maeyama 1986; Minagawa and Akazawa 1992; Chisholm 2004; Kobayashi, et al. 2004; Kusaka et al. 2010). Factors affecting regional variation in diet-related biomechanical stress include not only the foods eaten but also the way in which they are processed. There is archaeological evidence that the Jomon employed a range of sophisticated food processing techniques, from leeching and grinding acorns (Habu 2004; Kawashima 2016) to smoking and salting fish (Kobayashi et al. 2004; Habu et al. 2011; Robson et al. 2020). Such processes can transform the biomechanical properties of raw foods and must therefore be considered when inferring regional strain regimes. Hoover and Williams (2016) found that multivariate analyses of mandibular metrics differentiated between Jomon in Hokkaido, with their substantial reliance on marine mammal hunting, and more generalised fisher-hunter-gatherers in South/West Honshu. The authors attributed these morphological differences to reduced toughness in the South/West Honshu diet, potentially in tandem with less intense paramasticatory food-processing techniques in these regions (Hoover and Williams 2016). To date, the influence of dietary variation on Jomon crania, however, has not been investigated using geometric morphometric techniques, which preserve geometry and allow useful visualisation. Nor have they been investigated in a context explicitly and concurrently considering the effects of population history and climate as well as diet. The Jomon archaeological record is extremely rich and well-studied, allowing detailed reconstruction of subsistence practises and diets (Akazawa 1986a, 1986b, 1988, 1999; Akazawa and Maeyama 1986; Minagawa and Akazawa 1992; Kobayashi, et al. 2004). Here, we classify Jomon sites from different geographic regions by diet, based on their corresponding archaeology and inferred subsistence (see below and SI Sect. "Introduction").

If Jomon individuals can be grouped regionally by diet, and diet is a key factor in shaping Jomon crania, we would expect to see differences in cranial morphology between dietary groups. Hoover and Williams (2016) suggested that mandibular variation resulted from differences in biomechanical properties between regional diets. They found that the greatest difference in Jomon diet, and so in the inferred biomechanical forces shaping morphology, was between Hokkaido and South/West Japan. Following this logic, if differences in Jomon cranial shape found between geographic regions also reflect dietary strain, cranial differences in our sample should make sense in terms of the biomechanical properties of those diets, as inferred from the archaeology. Examples from previous research show morphology reflecting reduced dietary strain includes the shift to a shorter, more inferiorly placed temporalis muscle, neurocranial globularisation, smaller facial skeletons, and dental reduction, all of which are seen after the adoption of agriculture in multiple geographically-distant populations and which can be interpreted as a response to a softer diet (Katz et al. 2017; Menéndez and Buck 2022). If Hoover and Williams (2016) are correct, we would expect to see these types of differences between Hokkaido and South/West Jomon individuals, due to a softer biomechanical regime in the latter. Similarly, significant differences in estimated bite-force between our dietary groups would support the assertion that variation in dietary biomechanics was sufficient to influence Jomon cranial morphology (Menéndez et al. 2014). As mentioned above, differences between dietary groups that cannot be interpreted in the context of biomechanics may still be related to variation in the nutritional content of diets (Sardi et al. 2006; Menéndez et al. 2014), a possibility that we will discuss in the context of our results.

### Population history and cranial variation

Our second research question is: are there significant differences in cranial shape between Jomon sites that can be explained by the accumulation of stochastic variation as a result of population history? Stochastic genetic differentiation, resulting from neutral processes including mutation and drift, leads to the accumulation of between-group variation proportional to separation time between groups. This divergence between groups may also be shaped by factors such as gene flow and migration, resulting in greater complexity. As a result, patterns of neutral variation between groups are indicative of their population history. Population history has been shown to be the major explanatory factor for human cranial variation at a global scale (Roseman 2004; Roseman and Weaver 2004; Betti et al. 2009, 2010). If there is little contact between Jomon regions and build-up of random variation leaves a major signature on regional cranial variation, we should find an isolation by distance pattern such that nearby populations are similar to each other and distant ones differ substantially. Assessing the influences of both diet and population history on cranial morphology in this single Jomon sample will both ameliorate our understanding of how human phenotypes arise and also of this rich prehistoric forager culture.

Whilst the Jomon is considered a continuous culture (e.g., Habu 2004), the Japanese Archipelago is geographically complex, consisting of thousands of islands, high mountains, dense forest, and large rivers. These physical barriers, and most likely additional sociocultural ones, would have separated populations and allowed neutral genetic differences to accumulate. Research based on archaeology, bioarchaeology, and ancient DNA (aDNA) can shed some light on connectivity and group separation in Jomon-era Japan. The Jomon era is defined by its ceramics, the first of which usher in the Incipient Jomon period. In this early period, Jomon pots do not show marked regional differentiation, suggesting connectivity between different parts of Japan (Teruya 1986; Habu 2004). Later ceramic forms and styles of decoration, however, vary considerably between regions and change over time (Habu 2004; Kobayashi et al. 2004). By the Early Jomon period, regional styles are sufficiently divergent that some archaeologists have argued that they demonstrate the establishment of distinct groups of people (Habu 2004; Kobayashi et al. 2004). Kobayashi et al. (2004) argue that the smallest unit of ceramic style homogeneity that they recognise (‘nuclear style zones’) represents relatively autonomous groupings of Jomon people. They envisage these zones as encompassing populations with shared culture, social mores, and subsistence, resulting in a shared visual language expressed via ceramics. If this is the case, the authors assert that there would be little contact between people living in different nuclear zones. Yet, although some styles are localised to a single nuclear zone, others arise and then spread widely, suggesting connectivity at a larger scale (Kobayashi et al. 1992, 2004). Of course, it is difficult to tell from the presence of an artefact at a site whether it has itself travelled, either in a single step or by moving from person to person over multiple short distances, or whether the idea of how to make the artefact has travelled, but it at least implies a level of inter-group contact. Material analysis can elucidate this issue to some extent; petrological analyses show that about 70% of Early and Middle Jomon pots from sites in the Kanto region are made of local clay, suggesting local fabrication, but this changes in the Late period when local pots no longer form the majority of finds from the region (Habu 2004). Although typological and material analyses of Jomon ceramics are enlightening regarding Jomon population history, to date, their resolution is insufficient to generate clear predictions across regions and time periods (Kobayashi et al. 2004).

Archaeological finds of trade goods such as obsidian, jade, and asphalt, whose origin can be determined via materials analysis, demonstrate that they moved between regions during the Jomon era (Habu 2004; Kobayashi et al. 2004). Travel by people and movement of trade goods, at times over considerable distances, would have been facilitated by technology such as the dugout canoes uncovered at several Jomon sites (Habu 2004). Sub-tropical seashells from the Ryuku Islands in the far south of Japan, for example, have been found at sites as far north as Hokkaido (Habu 2004). These finds demonstrate the presence of distribution networks, although the actual movement of people and corresponding geneflow is harder to infer. Bioarchaeology and genetic analyses have been used to this end, to investigate regional relationships and connectivity between Jomon groups. Many Jomon individuals have striking ablation of their anterior teeth (Harunari 1986). Patterns of ablation (i.e., which teeth were removed) have long been suggested to be markers of group or individual social identity, perhaps even showing patterns of post-marital residence (Harunari 1986). Stable isotopic and morphological analyses attempting to confirm these hypotheses, however, are less than clear. At some Late-Final Jomon sites in the Kanto region, individuals shown by Strontium isotope analyses to be non-local are more likely to have a particular dental ablation pattern (albeit a pattern also found in locals) (Kusaka et al. 2011); however, the evidence from other similar sites seems to better support the suggestion that ablation patterns reflect aged-based familial groupings within resident communities (Temple et al. 2011). Whilst few nuclear aDNA analyses of Jomon remains have yet been carried out, analyses of ancient mitochondrial DNA (mtDNA) show a likely single origin for the Jomon and rapid dispersal throughout Japan (Adachi et al. 2021). Subsequently, there may have been limited maternal geneflow, i.e., limited female movement between regions, since later individuals seem to be heterogenous in their mtDNA haplogroups (Kanzawa-kiriyama et al. 2013; Adachi et al. 2013). Taken together, these diverse strands of evidence suggest a degree of separation existed between Jomon populations in different regions, overlain with routes of connectivity governed perhaps by trade and marriage networks. Degrees of disjunction or continuity appear to vary in space and over time, however, and as yet these patterns are insufficiently clear to be used to generate firm hypotheses regarding patterns of population history.

## Materials and methods

### Materials

#### Sample and data collection

**Fig. 1:**
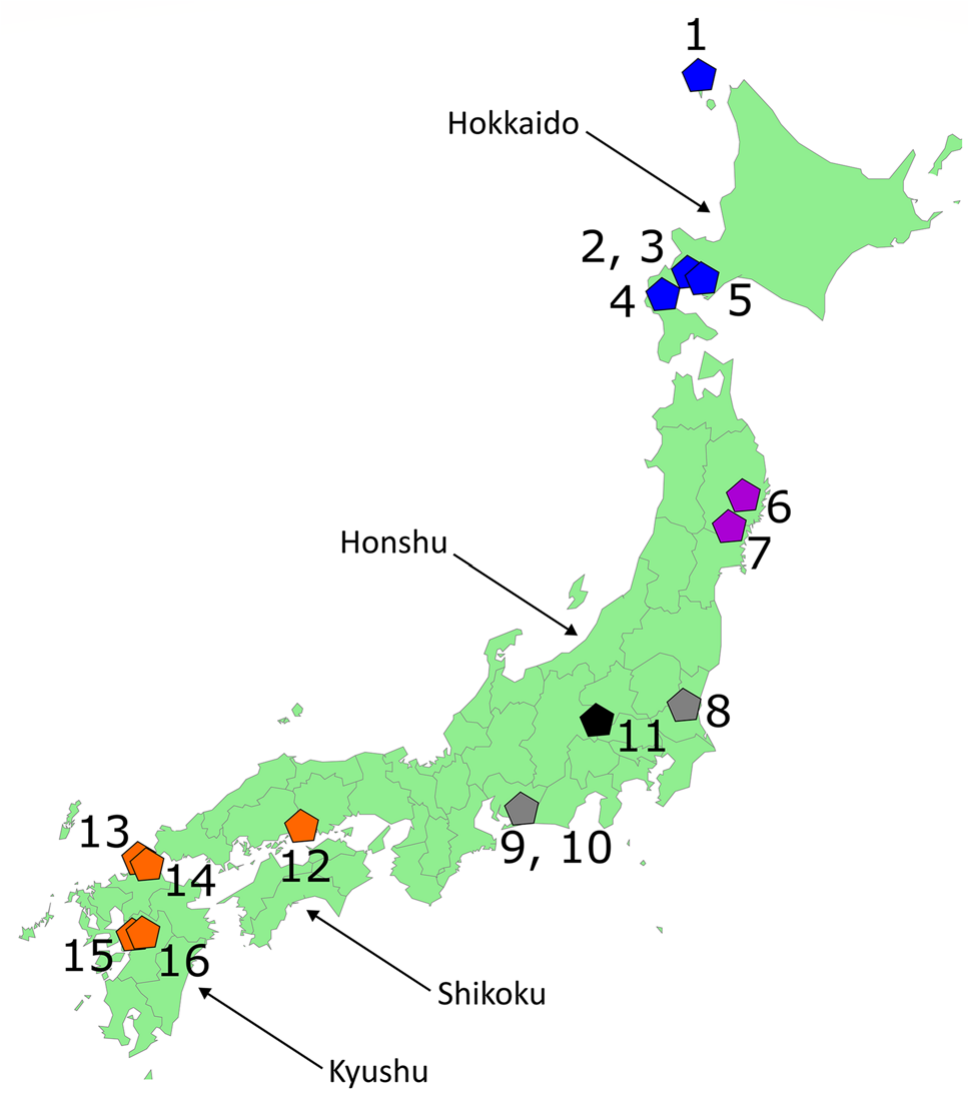
Map showing sampled Jomon sites and dietary regions used in this paper. The main islands of Japan (Hokkaido, Honshu, Shikoku, and Kyushu) are labelled. There are no Jomon from Shikoku included in this study. Coloured pentagons indicate dietary group; numbers indicate site. Dark blue: Hokkaido, purple: northeastern Honshu, grey: Coastal central Honshu, black: Inland central Honshu, orange: South/West Japan (Table 1). 1: Funadomari; 2: Takasago; 3: Irie; 4: Kotan Onsen; 5: Kitakogane; 6: Miyano; 7: Ebishima; 8: Wakaumi; 9: Yoshigo; 10: Ikawazu; 11: Tochibara; 12: Tsukumo; 13: Yamaga; 14: Einomaru; 15: Todoroki; 16: Goryo. Figure composed in Inkscape (Inkscape project, 2020)

Our sample consists of human crania from Jomon sites throughout the Japanese Archipelago (Fig. 1, Table 1, and Table S1) housed in the institutions listed in the Acknowledgements. For the specimens housed at Kyoto University, the crania were scanned by Satoshi Kobayashi on the university’s Alexion TSX-032A (Toshiba Medical Systems, Otawara, Japan). Scan parameters for individual specimens vary slightly with cranial size, but all voxel sizes are ~ 0.4 mm × 0.4 mm with a slice thickness of 1 mm. As CT scanning was not possible at the remaining institutions, LTB and BRH surface-scanned the majority of the remaining crania using an Artec Space Spider structured light scanner (Artec 3D, Luxenbourg) with a maximum resolution of 0.1 mm. We laser scanned a single individual, the cranium from Einomaru, with a Next Engine scanner (Next Engine, Santa Monica, USA) with a maximum resolution of 0.3 mm. We combined scanning methods to maximise our sample size, given time and financial constraints, and the need to study the specimens at their housing institutions. Analyses by other authors (e.g., Robinson and Terhune 2017; Shearer et al. 2017) suggest that the error introduced by combining different scanning methods is small relative to biologically meaningful signals in the data. We processed the surface scans in their respective proprietary software packages and exported the finished surface meshes to AVIZO (Thermo Fisher Scientific 2019). For the CT data, we exported surface meshes from isosurfaces in AVIZO. For all data modalities, landmark data were digitised in AVIZO on .ply surfaces. We collected five landmark sets (see SI Sect. "Materials and methods" and "Results" for full details) on subsamples of the Jomon crania, a craniofacial landmark set encapsulating overall cranial shape, three landmark sets focusing different cranial regions: facial, neurocranial, and temporalis regions, and a landmark set used to calculate bite-force. Different samples were used for each analysis to enable the inclusion of the maximum sample of these fragmentary archaeological remains in the study as a whole (Table S2).

**Table 1:** Sites in this study, with their allocated diet group, sample size, and key number in the map in Fig. 1

Site	Diet group	*n*	Map
Funadomari	HOK	5	1
Takasago	HOK	4	2
Irie	HOK	2	3
Kotan-Onsen	HOK	4	4
Kitakogane	HOK	4	5
Miyano	NEH	3	6
Ebishima	NEH	17	7
Wakaumi	CCH	1	8
Yoshigo	CCH	11	9
Ikawazu	CCH	4	10
Tochibara	ICH	3	11
Tsukumo	SWJ	20	12
Yamaga	SWJ	4	13
Einomaru	SWJ	1	14
Todoroki	SWJ	1	15
Goryo	SWJ	1	16

Note that not all individuals could be included in each analysis due to preservation issues (see Table S1). *HOK*, Hokkaido; *NEH*, northeastern Honshu; *CCH*, coastal central Honshu; *ICH*, inland central Honshu; *SWJ*, South/West Japan

#### Landmark data

**Fig. 2:**
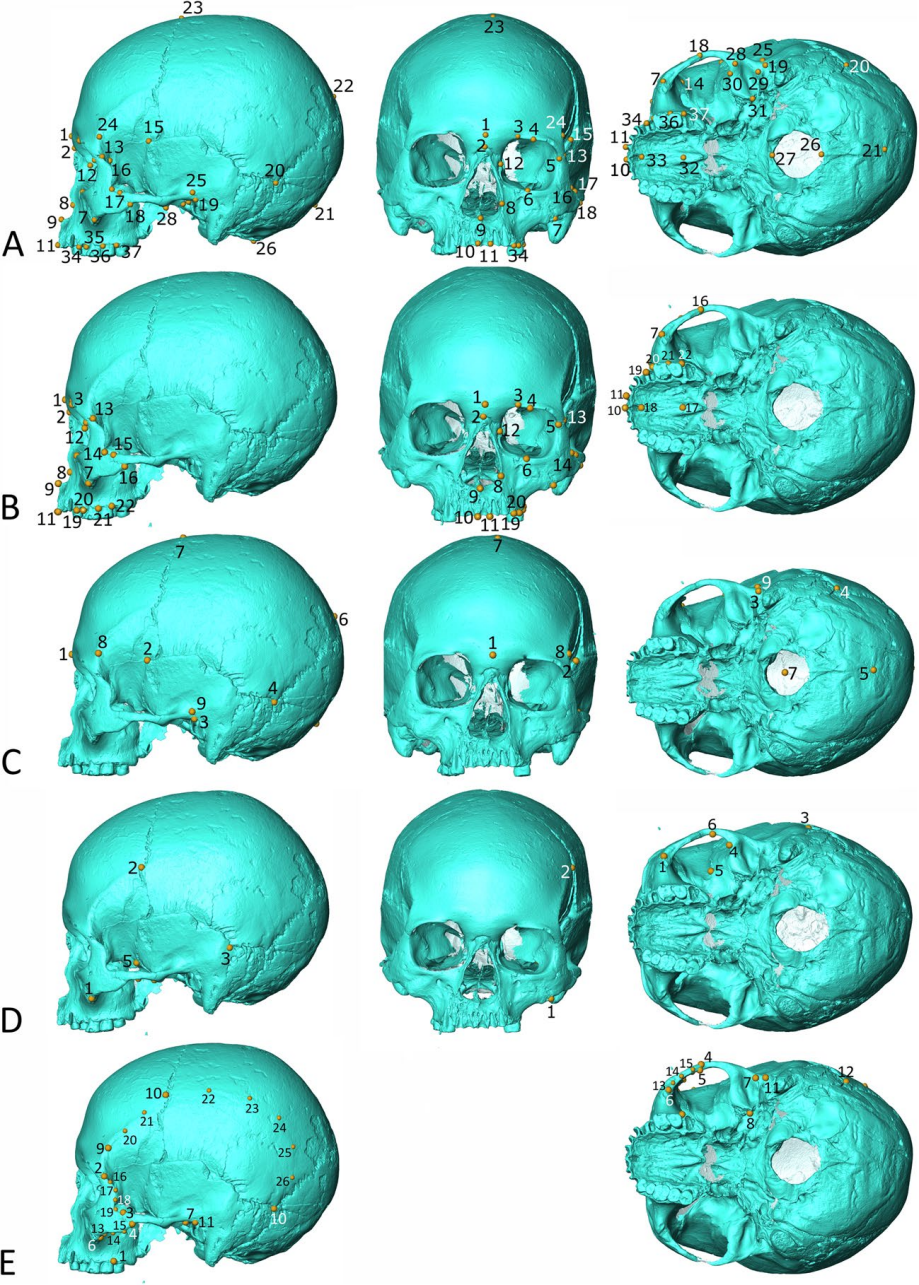
**A** craniofacial landmark set (Table S2); **B** facial landmark set (Table S3); **C** neurocranial landmark set (Table S4); **D** temporalis region landmark set (Table S5); **E** bite-force landmark set (Table S6); this landmark set is not shown from front because all landmarks are clearly visible in side or base views. In **A–D**, all landmarks are true landmarks; in **E**, landmarks 1–12 are true landmarks, and 13–26 are semilandmarks, denoted by smaller size. For explanatory text for D and E, see “Dietary groups” below. Figure composed in Inkscape using images from AVIZO

For a detailed breakdown of the sample for each analysis by site and sex, see SI Sect. "Materials and methods". The craniofacial landmark set (Table S2, Fig. 2) covers the entire external surface of the cranium with 37 landmarks and provides the best overall representation of cranial shape, but only allows the inclusion of 33 individuals due to poor preservation. The facial landmark set (22 landmarks; Fig. 2 and Table S3) is a subset derived from the craniofacial landmark set and as such only includes the same 33 individuals. We analysed facial shape separately because the face is the cranial region most likely to be affected by dietary stresses as it houses the maxillary teeth and many masticatory muscle attachments (Lieberman 2011). The neurocranial landmark set (Fig. 2 and Table S4) contains only nine landmarks but allows the inclusion of the greatest number of individuals (*n* = 83). The neurocranium is shaped in part by the attachment of the temporalis muscle, the largest and strongest muscle involved in mastication and it therefore may carry a dietary signal (Lieberman 2011; Noback and Harvati 2015; Menéndez and Buck 2022). Conversely, the neurocranium has been shown to carry a stronger signature of population history than the face and therefore this landmark data set may be most likely to demonstrate this influence (Harvati and Weaver 2006). Throughout this article, we use the term “cranial shape” to mean shape across these three landmark subdivisions (craniofacial + facial + neurocranial) and we refer to shape in those individual regions by name, e.g., “neurocranial shape”. In addition to the craniofacial, facial, and neurocranial landmark sets, we examined a set of six landmarks (Table S5, Fig. 2) reflecting the region of the temporalis muscle, to analyse the relationship between diet and the shape of the masticatory region in greater detail. This landmark set is based on landmarks previously shown to be effective in discriminating between related groups with different diets (Paschetta et al. 2010; Buck 2015; Menéndez et al. 2015). The preservation of the sample allowed us to include 60 individuals in the analysis of temporalis region shape. To investigate the relationship between the specific variable of dietary hardness and cranial morphology more explicitly, we also estimated bite force by using twelve landmarks and 14 semilandmarks (Table S6, Fig. 2) in 47 individuals, adapting the method proposed in Menéndez et al. (2014) (see below). In this analysis, the semilandmarks were used only to estimate muscle size, not shape, and the semilandmarks were not slid.

**Table 2:** Geographic distances between sites in km. Sites are ordered by numbers in Fig. 1

	Funadomari	Takasago	Irie	Kotan Onsen	Kitakogane	Miyano	Ebishima	Wakaumi	Yoshigo	Ikawazu	Tochibara	Tsukumo	Yamaga	Einomaru	Todoroki	Goryo
Funadomari																
Takasago	311.60															
Irie	312.72	2.57														
Kotan Onsen	319.25	34.68	32.22													
Kitakogane	327.87	18.86	19.28	45.16												
Miyano	685.03	377.91	377.36	381.57	360.03											
Ebishima	724.36	414.37	413.59	414.64	397.15	52.19										
Wakaumi	1025.69	714.10	713.00	709.35	697.96	355.23	307.57									
Yoshigo	1227.76	926.78	925.03	911.58	914.40	630.44	578.25	328.26								
Ikawazu	1228.30	927.41	925.66	912.16	915.05	631.40	579.20	329.50	1.32							
Tochibara	1049.08	743.05	741.45	730.44	729.63	436.05	383.86	171.15	194.41	195.36						
Tsukumo	1363.09	1095.31	1093.08	1071.37	1087.74	886.21	836.60	654.04	346.42	345.11	489.10					
Yamaga	1548.88	1306.10	1303.71	1278.75	1301.05	1137.98	1090.24	924.40	615.20	613.88	757.89	270.90				
Einomaru	1550.81	1306.81	1304.43	1279.61	1301.64	1136.23	1088.33	920.34	610.04	608.72	754.21	266.50	8.85			
Todoroki	1665.80	1413.60	1411.27	1387.56	1407.47	1220.82	1171.33	977.55	654.60	653.29	818.24	334.77	133.97	128.12		
Goryo	1660.06	1406.91	1404.59	1381.00	1400.69	1212.46	1162.90	968.09	644.90	643.59	809.05	326.30	133.18	126.84	10.10

**Table 3:** Pairwise comparison of Procrustes distances in neurocranial shape between dietary groups

	HOK	NEH	CCH	ICH	SWJ
HOK	-	0.191	0.930	0.693	0.099
NEH	0.026	-	0.177	0.628	< 0.001***
CCH	0.016	0.026	-	0.801	0.319
ICH	0.036	0.038	0.032	-	0.473
SWJ	0.025	0.038***	0.021	0.036	-

HOK, Hokkaido; NEH, northeastern Honshu; CCH, costal central Honshu; ICH, inland central Honshu; SWJ, South/West Japan. Matrix is symmetrical, below the trace: Procrustes distances, above the trace: *p* values from MANOVA. ***: significant at *p* < 0.001

**Table 4:** Pairwise comparison of Procrustes distances in temporalis region shape between dietary groups

	HOK	NEH	CCH	ICH	SWJ
HOK	-	0.402	0.004*	0.005*	0.229
NEH	0.032	-	0.007*	0.009*	0.134
CCH	0.073*	0.067*	-	0.031*	0.030*
ICH	0.114*	0.113*	0.118*	-	0.099
SWJ	0.040	0.045	0.067*	0.118	-

HOK, Hokkaido; NEH, northeastern Honshu; CCH, costal central Honshu; ICH, inland central Honshu; SWJ, South/West Japan. The matrix is symmetrical, below the trace: Procrustes distances, above the trace: *p* values from MANOVA. *: significant at *p* < 0.05

**Table 5:** Correlogram results showing Moran’s *I* and *p* values for craniofacial, facial, neurocranial and temporalis regions centroid size variation

Distance class	Mean distance (km)	Craniofacial size		Facial size		Neurocranial size		Temporalis region size	
		Moran’s *I*	*p*	Moran’s *I*	*p*	Moran’s *I*	*p*	Moran’s *I*	*p*
1	22.49	0.142	0.257	− 0.028	0.408	0.037	0.344	− 0.420	0.111
2	154.87	− 0.258	-	− 0.343	-	0.548*	0.014*	0.077	0.277
3	268.16	0.833	-	0.311	**-**	− 0.312	0.185	− 0.130	0.364
4	345.74	− 0.049	0.455	− 0.106	0.413	0.125	0.128	0.098	0.178
5	433.66	− 0.200	-	0.191	-	0.145	0.231	− 0.278	0.259
6	578.89	0.076	-	− 0.130	-	0.031	0.398	0.035	0.424
7	641.04	− 0.142	0.344	− 0.194	0.298	− 0.128	0.315	− 0.003	0.426
8	732.16	− 0.233	-	− 0.252	-	− 0.090	0.389	− 0.144	0.334
9	836.54	− 0.856	**-**	− 1.513	**-**	− 0.067	0.437	− 0.420	0.176
10	929.13	0.073	**-**	0.091	**-**	0.078	0.219	− 0.068	0.434
11	1075.11	− 0.067	**-**	− 0.008	**-**	0.016	0.392	− 0.133	0.383
12	1150.85	− 0.483	**-**	0.331	**-**	− 1.026*	0.028*	− 0.214	0.313
13	1241.25	0.393	**-**	0.451	**-**	0.388	0.072	− 0.114	0.437
14	1328.20	− 0.876*	0.029*	− 0.207	0.299	− 0.770*	0.016*	0.238	0.121
15	1407.43	1.094	**-**	1.502	**-**	− 0.424	0.121	− 0.054	0.443
16	1549.94	1.253	**-**	0.330	**-**	− 0.601	0.124	− 0.322	0.215
17	1663.42	− 0.772	**-**	− 1.718	**-**	− 0.190	0.286	0.438	0.153

In craniofacial and facial samples, there are some sites with no individuals, meaning that *p* values cannot be calculated for some distance classes. These are shown with “- ". *: significant results (*p* < 0.05)

**Table 6:** Correlogram results showing Moran’s *I* and *p* values for craniofacial, facial, neurocranial, and temporalis region shape (PC1 scores) variation

Distance class	Mean distance (km)	Craniofacial shape		Facial shape		Neurocranial shape		Temporalis region shape	
		Moran’s *I*	*p*	Moran’s *I*	*p*	Moran’s *I*	*p*	Moran’s *I*	*p*
1	22.49	0.402	0.115	0.640*	0.028*	0.059	0.295	− 0.155	0.416
2	154.87	0.109	**-**	− 0.724	**-**	− 0.421	0.155	0.885*	0.008*
3	268.16	0.125	**-**	0.032	**-**	0.545	0.119	0.111	0.297
4	345.74	− 0.092	0.484	0.025	0.330	− 0.276	0.163	0.009	0.321
5	433.66	0.072	**-**	0.133	**-**	0.337	0.139	− 0.104	0.380
6	578.89	0.133	**-**	− 1.799	**-**	− 0.137	0.369	0.116	0.298
7	641.04	− 0.159	0.337	− 0.865*	0.017*	− 0.370	0.086	− 0.591*	0.018*
8	732.16	− 0.467	**-**	− 0.112	**-**	0.128	0.233	− 0.187	0.267
9	836.54	− 0.260	**-**	0.783	**-**	− 1.155*	0.025*	− 0.572	0.113
10	929.13	0.423	**-**	− 0.110	**-**	0.335	0.049	0.059	0.270
11	1075.11	− 0.315	**-**	− 0.061	**-**	0.191	0.198	− 0.059	0.450
12	1150.85	− 0.551	**-**	0.889	**-**	0.283	0.190	0.091	0.300
13	1241.25	− 0.601	**-**	0.966*	0.017*	− 0.030	0.456	0.253	0.144
14	1328.20	− 0.260	0.316	− 0.281	0.271	0.366	0.066	− 0.151	0.347
15	1407.43	0.280	**-**	0.305	**-**	− 0.890*	0.034*	− 0.347	0.163
16	1549.94	− 0.262	**-**	− 0.041	**-**	− 0.073	0.437	0.110	0.288
17	1663.42	0.078	**-**	− 1.658	**-**	0.327	0.182	0.094	0.309

In craniofacial and facial samples, there are some sites with no individuals, meaning that *p* values cannot be calculated for some distance classes. These are shown with “- “. *: significant results (*p* < 0.05)

For each landmark set, we collected unilateral landmarks to remove the effects of bilateral asymmetry and to increase sample size by estimating missing bilateral landmarks preserved unilaterally. We digitised the most complete side of each cranium and then reflected all crania to the side of the first specimen in the sample as part of generalised Procrustes superimposition in MorphoJ (Klingenberg 2011). We used Morpheus (Slice 2005) to estimate missing landmarks by mirroring them across a midline calculated using all the other landmarks collected on that individual. Missing landmarks were estimated only where fewer than four landmarks were missing, to ensure accurate reflection using the imputed midline. We processed raw landmark coordinate data in MorphoJ and the R package geomorph (R Development Core Team 2015; Adams et al. 2020) and subjected them to generalised Procrustes superimposition to remove the effects of translation, rotation, and size. Following superimposition, we projected the landmark configurations into a tangent space to enable the use of Euclidean statistics.

#### Dietary groups

A classic body of research led by Takeru Akazawa (Akazawa 1982, 1986a, 1986b, 1988, 1999; Akazawa and Maeyama 1986) used geographic variation in Jomon material culture to classify technological regions and infer corresponding diets. Akazawa and colleagues first divided Japan into East and West; then, the eastern part was subsequently subdivided into three regions: the northeastern-most parts of Honshu and Hokkaido, a southeastern coastal part, and a more western part bordering the Sea of Japan and including inland eastern Japan (see SI for greater detail and a map). Their work shows that Jomon diet in these regions varied in terms of marine versus freshwater prey and differing reliance on vegetable and terrestrial foods (Akazawa 1982, 1986a, 1986b, 1988, 1999; Akazawa and Maeyama 1986). More recently, Hoover and Williams (2016) used a very similar scheme of regional dietary variation to Akazawa and colleagues’ to demonstrate a relationship between diet and mandibular morphology. The main difference between the Akazawa et al.’s dietary grouping and that of Hoover and Williams is that the latter separated out sites in Hokkaido from those in northeastern Honshu, due to the far greater reliance on marine mammals in Hokkaido (see also Oxenham and Matsumura 2008).

Here, we combine Akazawa and colleagues’ dietary groups with those of Hoover and Williams to classify our samples into the following dietary groups: Hokkaido, northeastern Honshu, coastal central Honshu, inland central Honshu, and South/West Japan (Table 1 and Fig. 1). In our combined scheme, based on the archaeology from sites in each region (Akazawa 1982, 1986a, 1986b, 1988, 1999; Akazawa and Maeyama 1986; Minagawa and Akazawa 1992; Oxenham and Matsumura 2008; Hoover and Williams 2016), the Hokkaido diet has a high marine component, with fishing of rocky coast and migratory fish and a substantial, specialised marine mammal component. The diet of the Jomon from the rocky coast of northeastern Honshu also shows heavy reliance on shellfish and migratory fish, but lacks the reliance on marine mammals seen in Hokkaido. Diet in the Kanto coastal lowland region around modern-day Tokyo Bay (here called costal central Honshu) is characterised by river, estuarine, and sheltered coastal resources. In the inland and Sea of Japan region of central Honshu (here: inland central Honshu), diet includes plant and freshwater resources, but is distinguished from other regions by greater hunting of terrestrial game. In South/West Japan, diet is characterised by exploitation of freshwater fish and a more substantial dependence on vegetable foods than in other areas.

### Methods

#### Preliminary analyses

We tested for the effects of sex and size on cranial, facial, neurocranial, and temporalis shape. To test the relationship between size and shape, we used the natural log of the centroid size of each landmark set as a proxy for size and used multivariate regression in R to test for its relationship with shape. Using the centroid size of each individual landmark set as a measure of size does not tell us about the relationship between cranial regions (e.g., the temporalis region) and overall cranial size, but the fragmentary nature of the sample necessitated different sample sizes for different samples, meaning that if we used craniofacial landmark set centroid size in the analysis of the relationship between temporalis region shape and size, we would have 60 configurations in the dependent variable and only 33 data points in the independent variable. The centroid size of the temporalis and masseter muscles is part of the equation used to calculate bite force metrics (see below), so the relationship between bite force and size was not measured separately but sex was also investigated for this variable. We determined sex designation in the sample by examining the pelvis (where possible) and cranium of each individual prior to scanning following standard osteological techniques. We classified crania in the sample as female, male, or uncertain (Table S2). We used Procrustes ANOVA on Procrustes coordinates in the geomorph package in R (Adams et al. 2020) to test for significant differences in shape between sex groups for each landmark set.

#### Analyses of diet

We classified specimens from the sites represented in our sample into dietary groups (Table 1 and Fig. 1) based on the technological and dietary classifications put forward by Akazawa and colleagues (Akazawa 1982, 1986a, 1986b, 1988, 1999; Akazawa and Maeyama 1986) and by Hoover and Williams (2016), as explained above. The only site in the inland central Honshu (ICH) group we could include was Tochibara (Fig. 1), with a maximum three individuals included, depending on analysis (Table S2). We tested the effect of including this very small sample by re-running analyses without it, but found it made very little difference (see SI Sect. 5), so we include the group in the results presented here for geographic and dietary completeness. We used MANOVA on Procrustes coordinates in R and Canonical Variates Analysis (CVA) in MorphoJ on craniofacial (*n* = 33), facial (*n* = 33), neurocranial (*n* = 83), and temporalis region landmark sets (*n* = 60) to evaluate the existence of differences amongst the regions and to characterise them. MANOVA tests for significant differences in shape amongst groups, whilst CVA maximises differences amongst those a priori groups to better visualise them. Here and throughout this study, results were judged to be significant if *p* < 0.05.

We calculated bite force by estimating the centroid sizes of the masseter and temporalis muscles and their lever arms, using the landmarks shown in Fig. 2 and Table S6, adapting the method used by Menéndez et al. (Menéndez et al. 2014). Landmarks 2–4 and 9–12 and semilandmarks 16–26 were used for temporalis estimation and landmarks 1, and 5–8 with semilandmarks 13–15 were used for masseter estimation. We used the 3D linear measurement tool in AVIZO to directly measure the moment arm on cranial surface meshes. The equation used to calculate bite force is $\mathbf{B}\mathbf{F}=\left[\left(\mathbf{T}\mathbf{C}\mathbf{S}\boldsymbol{*}{\varvec{t}}\right)+\left(\mathbf{M}\mathbf{C}\mathbf{S}\boldsymbol{*}{\varvec{m}}\right)\right]/\mathbf{M}\mathbf{o}$, where BF is the biteforce; TCS is the temporalis centroid size; *t* is the lever arm from the centroid of the temporalis to the TMJ; MCS is the masseter centroid size; *m* is the lever arm from centroid of the masseter to the TMJ; and *Mo* is the moment arm from the TMJ to the bite point (M2) (Menéndez et al. 2014). We compared bite force amongst dietary groups using ANOVA in the software PAST (Hammer et al. 2001), having first checked for normally distributed data using Shapiro–Wilk tests.

#### Analyses of population history

To explore the effect of population history on cranial variation amongst the samples, we calculated Moran’s* I* correlograms (see for example, Barbujani 2000; Perez and Monteiro 2009; Menéndez et al. 2014) using the “ncf” package in R (Bjørnstad and Falck 2001; R Core Team 2018) with size (centroid size) and shape (principal components scores for PCs 1 and 2) for each of the landmark sets (craniofacial, facial, neurocranial, and temporalis region). For these analyses, we used average values for each landmark set for each Jomon site and geographic distances which were calculated using decimal coordinates with the package “raster” in R (Hijmans 2015). Correlograms are biplots comparing an index of autocorrelation (Moran’s *I,* (Moran 1950)) against geographic distance (geographic distance classes). They allow the evaluation of whether pairs of data show spatial autocorrelation (i.e., the degree to which an object is similar to nearby objects) and the testing of hypotheses of randomness (i.e., if the pattern of variation observed is stochastic) for populations located at different geographic distances. The formula for Moran’s *I* is related to Pearson’s correlation coefficient; its numerator is a covariance matrix, comparing the values found at all pairs of data points in turn, whilst its denominator is the maximum-likelihood estimator of the variance (i.e., division by *n* instead of *n* − 1). Similarly, the significance of Moran’s *I* indicates the reliability of the autocorrelation index when considering a particular geographic distance amongst groups. When Moran’s *I* values are close to 0, it typically indicates no spatial autocorrelation; thus, the observed variation is randomly distributed across samples located at a particular geographical distance. Positive Moran’s *I* values indicate positive spatial autocorrelation, meaning that groups that are located at a certain distance class present similar cranial shapes or sizes, whilst negative Moran’s *I* values result from groups that differ widely in cranial shape and/or size. In a classic isolation by distance pattern, such as that seen in worldwide cranial variation due to the ‘Out of Africa’ signal (Relethford 2004), one would expect an asymptotic decrease, a transition from more positive Moran’s *I* values, as a result of a high spatial autocorrelation at the first distance classes, towards zero values indicating random variation with increasing geographical distance (Barbujani 2000). Decimal coordinates of latitude and longitude are used to calculate geographical distances in km and to calculate distance classes used in the correlograms. In the current paper, we selected seventeen equally sized classes, each one ranging 100 kms (e.g., distance class 1 = 0–100 km, includes all sites located < 100 km from each other). For instance, distance class 1 represents groups that are located at an average of 22 km from one another and thus includes the pairs presenting the lowest distances from all the samples, e.g., Einomaru to Yamaga (~ 9 km) in Kyushu, South/West Japan, and Kitakogane to Takasago (~ 19 km) in Hokkaido (Table 2).

## Results

### The effects of size and sexual dimorphism

A small portion of the shape variation amongst the samples can be explained as a result of differences in size. A Procrustes ANOVA of craniofacial shape on Ln craniofacial centroid size is significant (*F*(1, 31) = 1.47, *r*^2^ = 0.045, *p* = 0.04) and explains 4.53% of shape variation. Likewise, Procrustes ANOVA shows that there is a significant relationship between temporalis shape and Ln temporalis region centroid size, with size explaining 4.27% of shape variation in the temporalis region (*F*(1,59) = 2.63, *r*^2^ = 0.043, *p* < 0.05). In neither the craniofacial nor temporalis region analysis is there a clear difference amongst dietary groups in the relationship between size and shape (see SI Sect. 6), although in the case of the craniofacial analysis this determination is hampered by the low sample size in some of the dietary groups. The relationships between facial shape and facial Ln centroid size and between neurocranial shape and neurocranial Ln centroid size are both non-significant (*p* > 0.05).

**Fig. 3:**
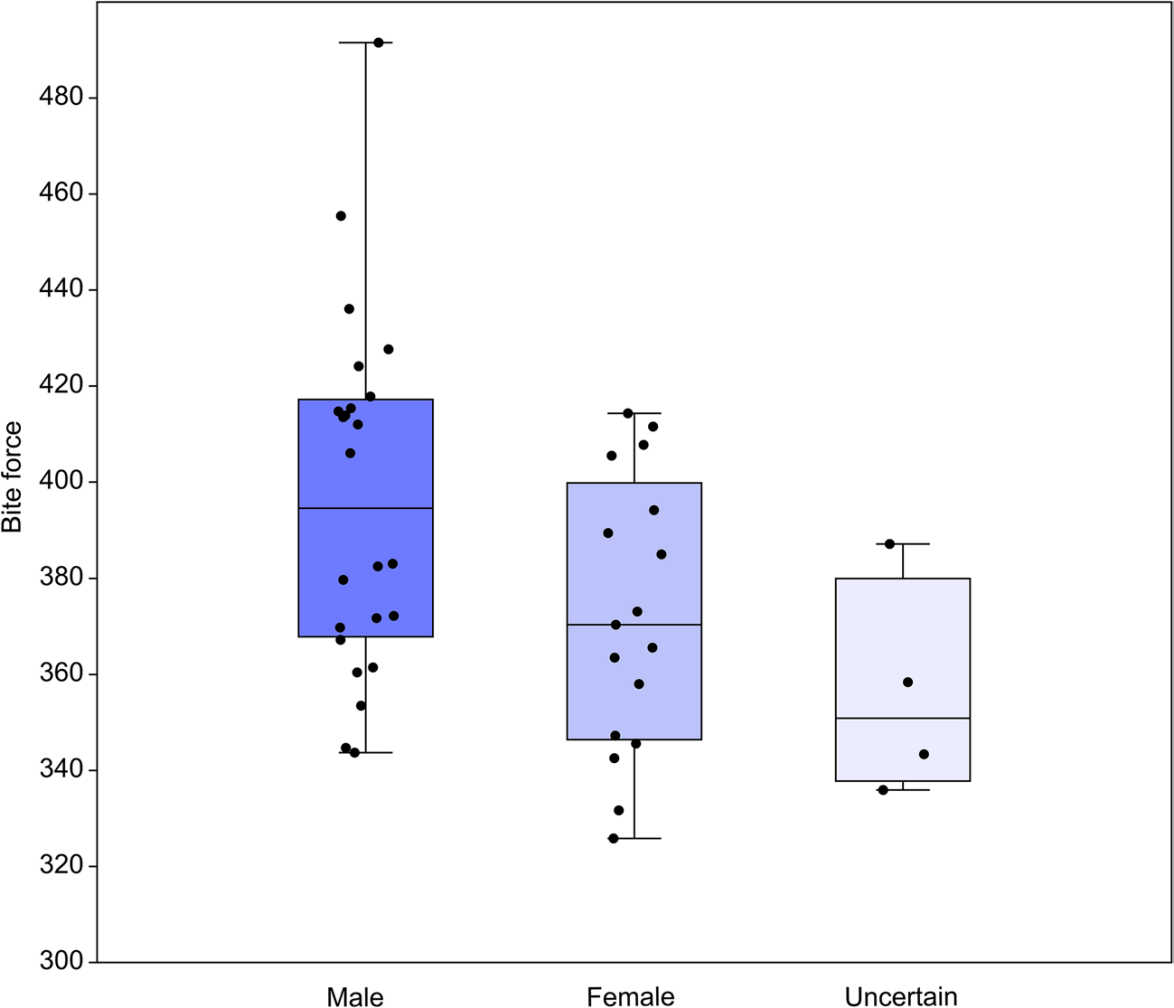
Boxplot with jitter showing bite force by sex. Figure created using PAST and edited for aesthetics using Inkscape

Regarding sexual dimorphism, there are no substantial shape differences amongst the sexes in any analysis in this sample: Procrustes ANOVAs of cranial (*F*(2,32) = 0.76, *p* > 0.05), facial (*F*(2,30) = 1.14, *p* > 0.05), neurocranial (*F*(2, 80) = 1.52, *p* > 0.05), and temporalis region (*F*(2, 58) = 0.68, *p* > 0.05) shape, all show no significant differences. There are significant differences in the calculated bite force values between the sexes (ANOVA: *F*(2, 42) = 4.21, *p* < 0.05), although in Tukey’s post-hoc tests, none of the individual comparisons between sexes reaches significance (*p* > 0.05). Examination of Fig. 3 shows that bite force tends to be greater in males than females or individuals of uncertain sex but that there is overlap between the majority of the ranges for each sex. The complete overlap between female and uncertain samples precludes the removal of the latter group to obtain better discrimination between males and females (see also SI Sect. 6). Based on these results, we conclude that the effects of size and sexual dimorphism on the variables under study within this sample is small to negligible and so, to maintain sample sizes, we pool the sexes for subsequent analyses.

### Differences between dietary groups

**Fig. 4:**
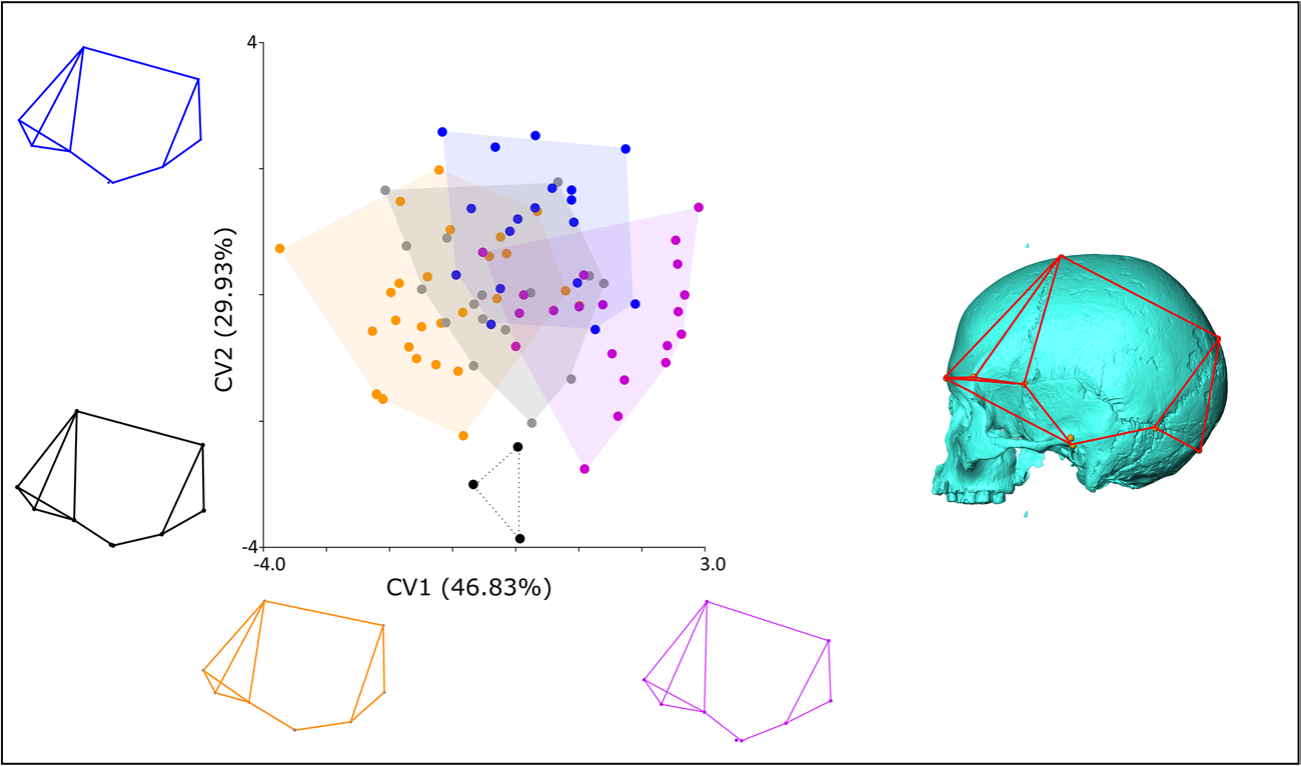
Left: CVA showing neurocranial shape variation amongst dietary groups. Dark blue: Hokkaido; purple: northeastern Honshu; grey: coastal central Honshu; black: inland central Honshu; orange: South/West Japan. Wirefames (see right-hand image) show extremes of respective CVs in lateral views. Figure composed in Inkscape using images from MorphoJ and AVIZO

MANOVA shows there are no significant differences in craniofacial (*F*(4,28) = 1.09, *p* > 0.05) or facial (*F*(4, 28) = 1.22, *p* > 0.05) shape between dietary groups; however, there is a significant difference in neurocranial shape (*F*(4,78) = 1.60, *p* < 0.005). CVA with a permutation test of significance of Procrustes distances between groups shows there is a significant difference between crania from northeastern Honshu and South/West Japan (Table 3) with the groups well-separated on CV1, which explains 46.83% of group differentiation (Fig. 4). The other diet groups are intermediate on this CV. Jomon from South/West Japan configurations mostly fall towards the minimum of CV1, compared to northeastern Honshu configurations, which mainly fall towards the maximum (Fig. 4). The neurocranial shape of individuals towards the minimum of CV1 is more uniform in superoinferior depth from anterior to posterior, with a superoinferiorly shorter, flatter inferior neurocranial portion as shown by the smaller distances between both pterion posterior (2) and asterion (4) and porion (3). The anterior portion of the neurocranium (as delimited by bregma (7) superiorly, glabella (1) anteriorly, frontotemporale (8) laterally, and pterion posterior (2) posteriorly) is smaller in individuals at the lower end of CV1, particularly in the superoinferior and anteroposterior dimensions. The occipital region in individuals at the lower end of CV1 is superoinferiorly taller and narrower mediolaterally at asterion (4) and the angle at inion (5) is more obtuse (Fig. 4). There is also an interesting distinction (Fig. 4) on CV2 (29.93% group differentiation) between the three individuals from the inland central Honshu diet group and the Hokkaido diet group, but this does not reach significance in the MANOVA, likely due to the very small number of individuals in the former group (Table 1). When compared to Hokkaido individuals, inland central Honshu individuals falling at the minimum of CV2 are differentiated by an anteroposteriorly longer and mediolaterally narrower neurocranium. Individuals from this group have an anteroposteriorly longer and more anteriorly angled anterior portion of the neurocranium, with bregma placed more superoanteriorly. The posterior portion of the neurocranium (as delimited by lambda (6) superiorly, inion (5) posteriorly and asterion (4) laterally) is more posteriorly placed and is longer anteroposteriorly.

**Fig. 5:**
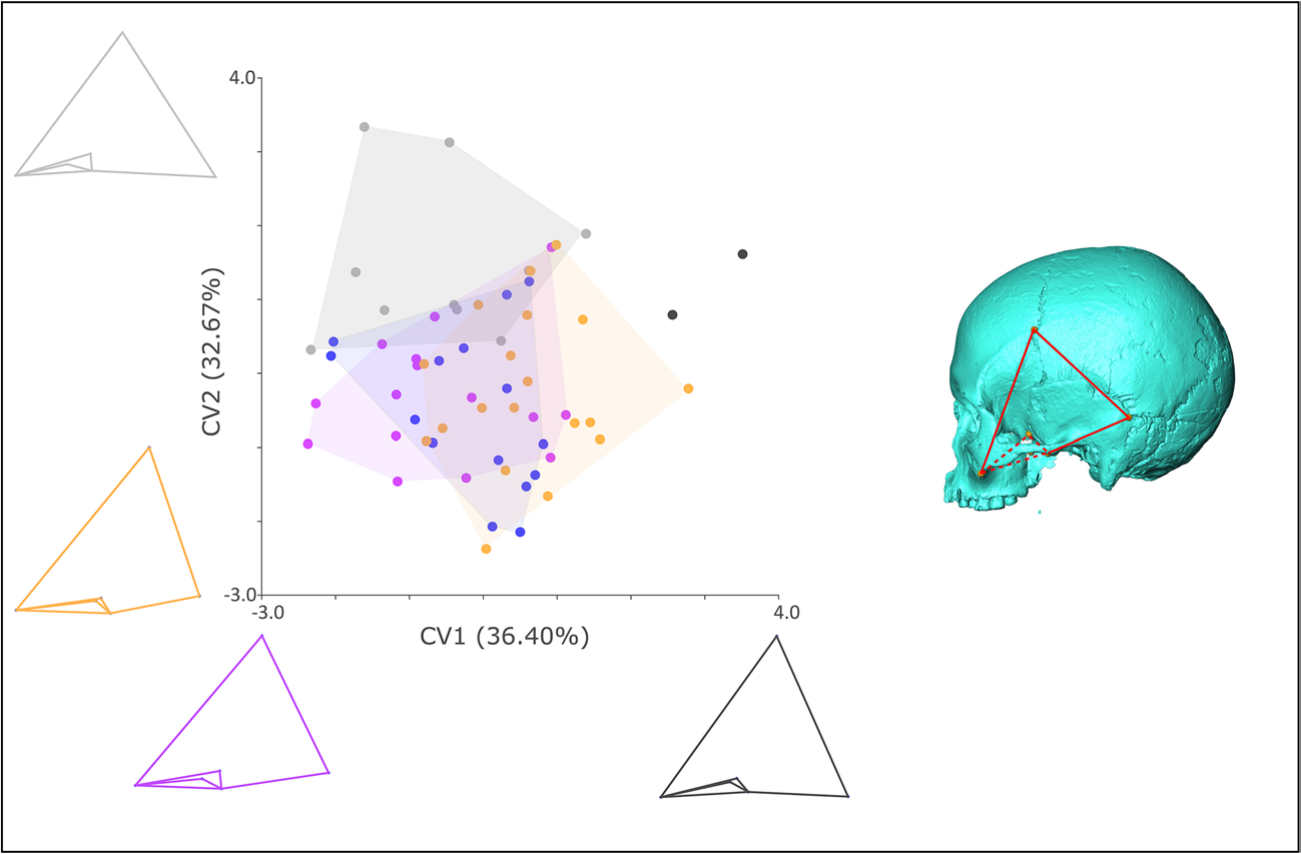
Left: CVA of temporalis shape amongst dietary groups. Dark blue: Hokkaido; purple: northeastern Honshu; grey: coastal central Honshu; black: inland central Honshu; orange: South/West Honshu. Wirefames (see right-hand image) show extremes of respective CVs in lateral views. Figure composed in Inkscape using images from MorphoJ and AVIZO

In the analyses of temporalis region shape, MANOVA shows that there is a significant difference in shape between dietary groups (*F*(4,78) = 1.60, *p* < 0.005). CVA with a permutation test of significance for differences in Procrustes distances between diet groups shows that coastal central Honshu is significantly different from all groups and inland central Honshu is significantly different to all groups except South/West Japan (Table 4). Visualisation of CV1 (36.40% variance) shows that the South/West Japan group tends to score more highly than the other groups, with the two inland central Honshu individuals at the extreme of this variation and beyond it (Fig. 5). The higher end of CV1 is distinguished by a more upright, anteriorly placed temporalis region in lateral view. Stephanion (2) is more superoanteriorly placed and zygomaxillare (2) and ethomion (3) more inferoanteriorly placed, compared to the rest of the configuration. The superior border of the temporal fossa, delimited by landmarks MW1 (5) and MW2 (6), is also relatively superior compared to configurations towards the lower end of CV1. When viewed inferiorly, high-scoring configurations on CV1 show a considerably narrower temporal fossa across MW1 and MW2. Although inland central Honshu individuals represent the highest extreme of CV1, there are only two individuals from this group in the temporalis region analysis, so this result should be treated with caution. On CV2 (32.67% variance), inland central Honshu and particularly coastal central Honshu are relatively well separated out from the other groups at the upper end of the CV, although there is some overlap with all of the other groups towards the mid-range of the CV (Fig. 5). Configurations at the upper end of CV2 show temporalis regions that are considerably shorter superoinferiorly and longer anteroposteriorly, extending further inferoposteriorly at ethomion. In these individuals, the temporal fossa is more superiorly placed and is much broader mediolaterally (Fig. 5).

**Fig. 6:**
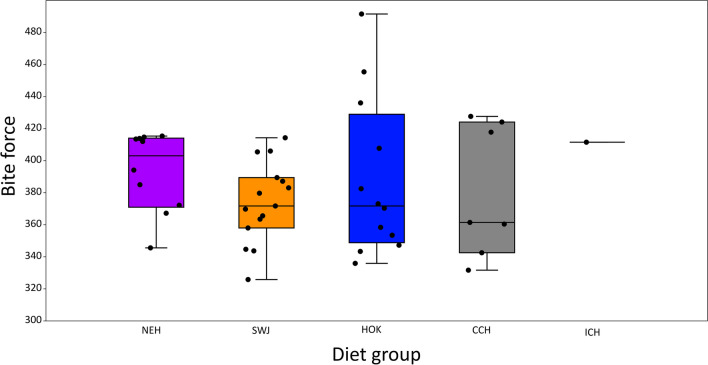
Boxplot with jitter showing bite force by dietary group. NEH, northeastern Honshu; SWJ, South/West Japan; HOK, Hokkaido; CCH, coastal central Honshu; ICH, inland central Honshu. Figure created using PAST and edited for aesthetics using Inkscape

The bite force data are normally distributed (Shapiro Wilks tests show *p* > 0.05), and an ANOVA shows no significant difference in bite force amongst dietary groups (Welch *F* test used due to significant Levene’s test for homogeneity of variance (*p* < 0.05). *F* = 1.19, df = 17.7, *p* > 0.05). The sole inland central Honshu individual for which it was possible to calculate bite force (Tochibara KA1) was not included in this ANOVA as > 1 individual is required in each group. Figure 6 shows that there is a trend for lower bite forces in South/West Japan compared to the other groups, but that there is considerable overlap between all groups. There is substantial variation in Hokkaido bite forces; one individual, Takasago 133, has a particularly high bite force but removing this specimen does not affect the results. Individuals in the coastal central Honshu group appear to fall into two groups, but these groups are not patterned by site or sex (Table 5).

### Population history

**Fig. 7:**
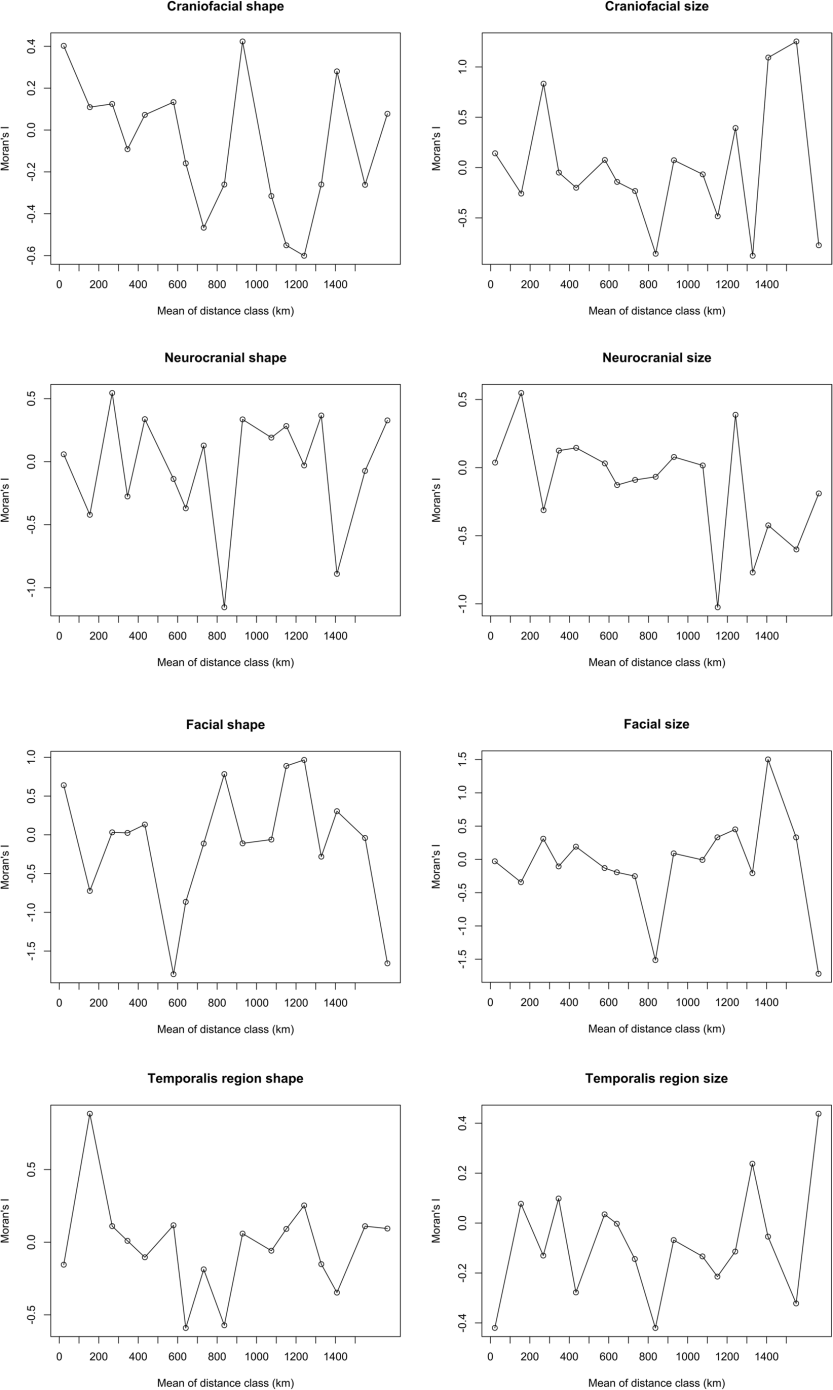
Correlograms showing spatial autocorrelation in size (left column; PC1 scores) and shape (right column; centroid sizes) between sites for different landmark sets. Figures created in R

We did not find any geographic pattern of variation amongst the sites, instead, morphological variation varies randomly in all cases. The correlograms in Fig. 7 show variation in shape, represented by PC1, and size, represented by centroid size for each cranial module. We also ran the correlograms for PC2 and the results (not shown) do not differ from PC1. The first distance class (mean distance between sites: ~ 22 km) shows the variation amongst populations that are located near to one another. Surprisingly, only craniofacial and facial shape is relatively similar, though not significantly so, amongst individuals from sites that are close to each other (Fig. 7 and Table 6). On the contrary, the temporalis size differs amongst the neighbouring sites (Fig. 7 and Table 5), whilst the other cranial modules show random variation amongst nearby sites. The last distance class (mean distance ~ 1663 km) shows in general Moran’s *I* values close to or below zero as expected for random variation or large differences amongst individuals from distant sites. The exception to this is the temporalis size analysis, which presents moderate similarities between sites in the maximum distance class, although this is not significant (Fig. 7 and Table 5).

## Discussion

### Differences in cranial shape between dietary groups

We found no significant differences in craniofacial or facial shape between dietary groups, suggesting either that variation in diet does not influence inter-group variation in overall and facial shape substantially, or that our samples/landmark data are insufficient to capture this signal. Neurocranial shape, however, differs significantly between dietary groups. The neurocranial analysis included the largest sample of any analysis (*n* = 83), yet only nine landmarks. Significant differentiation with data providing such broad shape characterisation may indicate the strength of group dissimilarity. Our results show discrimination between the northeastern Honshu and South/West Japan groups. This reflects a fundamental divide in Japanese ecology to this day, which is evident in the deciduous forest in the northeast and evergreen laurel forest in the southwest of the archipelago (Yasuda 1978; Akazawa 1982; Tsukada 1986). The northeast/southwest biogeographic divide is evident in the morphology (Buck et al. 2018, 2019) and genetically-determined phylogeny of Japanese macaques (Ito et al. 2021), suggesting that environment plays a role in population structure and cranial adaptation in these non-human primates. Such a relationship was not apparent in our earlier ecogeographic study of the Jomon, however, which concentrated on the relationship between cranial morphology and climate (Buck et al. 2019). Similarly, it does not seem to reflected in Jomon population structure as reconstructed from mtDNA (Mizuno et al. 2023), although similar nuclear DNA studies have not yet been possible. This suggests that climate and population history are not leading to the patterning in neurocranial shape seen in this study (see below for a discussion of population history in this study). It has long been argued, however, that this ecological divide translates into differences in Jomon material culture, including that relating to subsistence activities (Yasuda 1978; Akazawa 1986a; Kobayashi et al. 2004), and it is thus one of the major divisions in diet described by Akazawa (1986a). The most successful discrimination between diet groups in Hoover and Williams’ (2016) analysis of mandible morphology was between Hokkaido and South/West Honshu. It seems plausible, therefore, that our results are capturing a similar dietary signal in the neurocranium. Although we see no significant difference between Hokkaido and South/West Japan, in contrast to Hoover and Williams, in our results Hokkaido does overlap almost entirely with northeastern Honshu on CV1, the axis explaining greatest variation in neurocranial shape within the sample (Fig. 4). The lack of differentiation in this study between northeastern Honshu and Hokkaido fits better with Akazawa and colleagues’ dietary divisions than those of Hoover and Williams. Perhaps the shape of the mandible, which reflects diet more faithfully than the cranium (von Cramon-Taubadel 2011), picks up on more subtle differences between these two northern dietary groups, such as the prevalence of marine mammals in the Hokkaido diet.

In the current study, mean South/West Japan Jomon neurocranial shape is significantly differentiated from that of northeastern Honshu Jomon, with other groups falling intermediately and not significantly separated from one-another. There are potential biomechanical explanations for these differences in shape. South/West Japan individuals tend to have a more constant superoinferior height throughout the neurocranium from anterior to posterior, which gives these neurocrania a more globular appearance in the sagittal plane (Fig. 4). A more globular neurocranium is one of the cranial hallmarks of the shift from a forager diet to an agricultural one in several geographic regions. It is ascribed to reduced action of the masticatory muscles on the neurocranium in a less demanding dietary regime resulting from agricultural subsistence (for a review, see Menéndez and Buck 2022). In contrast to the South/West Jomon, the neurocrania of the northeastern Honshu group tend to have high, large frontal regions and low, more compressed and angled occipital regions (Fig. 4). The superoinferiorly taller and mediolaterally wider frontal regions could correspond to a larger region for temporalis muscle attachment in the more northern Jomon (see below). A larger, more anteriorly placed temporalis is widely thought to relate to a diet high in masticatory strain and this morphology is seen in many forager populations when compared to agriculturalists who are assumed to have diets with reduced biomechanical requirements (Lieberman 2011; Noback and Harvati 2015; Katz et al. 2017; Menéndez and Buck 2022).

To recap, in the northeastern Jomon diet, there was an inferred greater reliance on marine fisher-foraging than in the broader-spectrum foraging of the South/West of Japan (Akazawa 1986a; Akazawa and Maeyama 1986). The Pacific coasts of northeastern Japan are very rich fishing grounds due to the meeting of the Oyashio and Kuroshio currents (Akazawa and Maeyama 1986; Akazawa 1988), and the Jomon diet of this region was characterised by the exploitation of large fish, particularly migratory species (Akazawa 1986a, 1988). In the South/West of Japan, Jomon people inhabited mosaic ecosystems of freshwater sources and laurel forest (Akazawa 1986a). The archaeology from sites in this region shows a considerable reliance on plant foods, including querns, grinding stones, and chipped stone axes for mechanical processing and pits for nut storage (Akazawa 1986a, 1986b, 1999; Akazawa and Maeyama 1986). Isotopic evidence from Yosekura (Chugoku, South/West Japan in the dietary scheme used here) corroborates the archaeological record, showing a signature similar to that of large herbivores from the region (Akazawa and Maeyama 1986) demonstrating the high proportion of vegetable foods in South/West Jomon diets.

How such dietary differences between South/West Japan and northeastern Honshu may have translated into masticatory requirements is less straightforward, however. Authors discussing the impact of agriculture often suggest that wild meats, tough plants, tubers, and nuts are sources of greater dietary stress than farmed foods (see Menéndez and Buck, 2022, for a review), but such properties are complex and dependent on many factors, particularly preparation techniques (Zink et al. 2014). These factors are harder to unpick when comparing amongst Jomon forager diets, which were all essentially broad-based, but which were composed of varying proportions of different wild food types. Experimental testing of foods in the Jomon diet is needed to make firm conclusions about differing biomechanical stress regimes between regions, but some suggestions can be made. Many of the key plant foods in the Jomon diet, such as horse chestnuts, acorns, tubers, and some mountain vegetables, require considerable processing to make them edible or palatable (Habu 2004; Kobayashi et al. 2004; Kawashima 2016). Plants formed a particularly important part of the diet in the South/West Japan region and the archaeological record from South/West sites is particularly characterised by technology enabling processes such as boiling, soaking, pounding, and grinding, which would render vegetable foods soft (Akazawa 1986a, 1986b, 1999; Akazawa and Maeyama 1986; Habu 2004). Thus, greater reliance on vegetable foods in the South/West Japan group might lead to a proportionally less biomechanically demanding diet. Conversely, in northeastern Honshu, there is evidence for a strategy of seasonally intensive fishing and shellfish collecting (Akazawa 1986b). Techniques for preserving fish and shellfish thought to be practised by the Jomon to extend its life beyond its seasonal abundance, such as drying, smoking, and salting, increase its toughness so that a diet where this was a more important resource may have been more biomechanically demanding (Habu 2004; Kobayashi et al. 2004; Hoover and Williams 2016; Kawashima 2016, 2022). As Hoover and Williams (2016) have suggested, paramasticatory activity in the preparation of hides and/or the production of complex fishing and marine hunting tools, which are particularly evident in this region (Akazawa 1986a, 1988), may have been an additional source of strain for northeastern Jomon. Paramasticatory strain has previously been suggested to be an important influence on craniofacial shape for more recent high-latitude foragers such as Inuit people (Hylander 1975). We can then tentatively suggest that the northeastern Honshu diet may have been characterised by higher strains than the South/West Japan diet, which contributed to the differences in neurocranial shape between the groups.

In our analyses of temporalis muscle region, shape also varies significantly between dietary groups. In this analysis, the central two groups are most distinct; central coastal Honshu is significantly different from all other groups and inland central Honshu is significantly different from all other groups except South/West Japan. As there are only two individuals from inland central Honshu included, however, it would be unwise to consider them alone. When individual axes of variation maximising group differentiation are considered using CVA, the greatest group differentiation is between South/West Japan and inland central Honshu on the one hand and coastal central Honshu, northeastern Honshu, and Hokkaido at the other (Fig. 5). The temporalis region shape difference described by this division is between an anteroposteriorly shorter, superoinferiorly taller temporalis region with a mediolaterally narrower temporal fossa in South/West Japan and inland central Honshu, and an anteroposteriorly longer, superoinferiorly shorter temporalis region with a mediolaterally wider temporal fossa in coastal central Honshu, northeastern Honshu, and Hokkaido (Fig. 5). This corresponds to a difference between more plant-heavy diets in the former southern and inland groups and more marine-heavy diets in the coastal and northern groups. Biomechanically, this could be interpreted in a similar fashion to the neurocranial results. If a more plant-based diet requires greater food processing, softening many tough foods, and a diet with high proportions of smoked and preserved fish results in high masticatory strains, the South/West Japan and inland central Honshu diets could be less biomechanically demanding than the coastal central Honshu, northeastern Honshu, and Hokkaido diets. This could have led to a more globular cranium in the southern and inland groups, as reflected in the taller, more upright triangle-shape of the temporalis regions in these individuals. This is a pattern observed when farmers and foragers are compared across global samples (Menéndez and Buck 2022). Similarly, a thinner body of the temporalis muscle, as reflected in the narrower temporal fossa in the southern and inland groups (Fig. 5), is in keeping with farmer/forager comparisons, which have shown that foragers, with more biomechanically demanding diets, tend to have larger and thicker temporalis muscles than farmers (Noback and Harvati 2015).

The differentiation of coastal central Honshu from the two more northerly groups (Hokkaido and northeastern Honshu) on the second canonical variate is less easy to explain biomechanically. Although in both instances the temporalis regions tend to be anteroposteriorly longer and superoinferiorly shorter than in SWJ/ICH, in coastal central, Honshu there appears to be greater extension of the temporalis region inferoposteriorly, whereas in Hokkaido and northeastern Honshu individuals, the extension is greater inferoanteriorly. More sophisticated modelling of temporalis morphology and action, such as finite element analysis could shed light on this matter, but these samples are in any case relatively small and may be confounded by other factors such as chronology, social status, or stochastic variation.

The relationship between food-types and their biomechanical properties in an archaeological sample of foragers with very diverse diets is not straightforward. We therefore estimated bite force as a proxy for the actual mechanical strains experienced by the Jomon groups during mastication. Contrary to the suggestions of neurocranial and temporalis region shape described above, in the current sample, we found no significant differences in bite force between dietary groups. This suggests there may have been insufficient differences in dietary hardness for morphological differentiation to result, hard foods being those with high stiffness that require high bite forces to fracture them during mastication (Lieberman 2011). We estimated bite force using the size and lever arms of the masseter and the temporalis, using M^2^ as the bite point (see Methods). Whilst the masseter and temporalis are the largest masticatory muscles, it is possible that estimating the biomechanical properties of other muscles, such as the medial pterygoid, or other bite points along the toothrow could have provided additional useful information in teasing apart regional biomechanical regimes. It would be profitable to investigate this in future work, ideally also including the mandible where it is preserved. In addition to hardness, there are other food properties that may also affect the biomechanical link between diet and skull morphology. The relationships between the different food properties such as hardness or toughness, bite force, and morphological variables such as masticatory muscle, and skull, size, and shape, are incompletely understood (Berthaume 2016). In the anthropological literature, these issues have been considered most thoroughly in the context of non-human primates (e.g., Corruccini and Beecher 1982; McGraw and Daegling 2020; Panagiotopoulou et al. 2020) and potentially hard-object feeding megadont hominins such as *Paranthropus* (Strait and Grine 2004; e.g., Dominy et al. 2008; Smith et al. 2015), with less application to populations of *Homo sapiens* with different diets. It is possible that most of the foods eaten by different Jomon groups are of too similar hardness to elicit differentiation in bite force capacity, in contrast to food sources such as un-shelled nuts which seem to lead to marked adaptations in other species (Wright 2005). Conversely, the regional variation in muscle size and shape we infer from our results could perhaps be associated with other dietary properties, such as a greater dietary toughness in a diet with less processed vegetable foods and increased preserved marine sources of protein. In order to distinguish these possibilities, better characterisation of the biomechanical properties of food sources and food preparation techniques in regional Jomon diets is needed to determine how a diet rich in marine mammal meat, shellfish and sea fish might differ from one with an emphasis on terrestrial game and plant foods. Analyses of foods potentially forming a component of australopithecine diets provide a guide for how this might be achieved (see for example, Dominy et al. 2008).

In addition to the biomechanical properties discussed above, other dietary variables may influence cranial development. Differences in bite force do not explain craniofacial variation amongst South American populations with different diets, but distinct protein and carbohydrate proportions in diets have a strong impact on cranial morphology (Sardi et al. 2006; Menéndez et al. 2014). Experimental work on non-human species shows that hormonally controlled growth decreases in the absence of sufficient protein (Dressino and Pucciarelli 1999; Cónsole et al. 2001), and a similar mechanism has been suggested for the reduction in body size in prehistoric South African humans during their adoption of agriculture (Ginter 2011). There is no clear relationship between size and dietary group in the current sample, so that systematic size reduction due to insufficient protein would not explain our results; however, nutritional deficiency not only leads to smaller size but also to concomitant allometric shape changes (Menéndez et al. 2014), which may be more complex to interpret. The archaeology of Japan argues for a greater plant component to diets in inland central and southern/western regions (Akazawa 1986a, 1986b; Akazawa and Maeyama 1986), but more carbohydrates do not necessarily mean insufficient protein. The inland and southern/western regions also show evidence for freshwater aquatic resources and, particularly in the inland central Honshu region, terrestrial game was hunted (Akazawa 1986a). It is not clear that dietary differences in the order of a greater reliance on marine mammal meat, compared to estuarine fish, compared to terrestrial game, would translate into variation in nutritional composition capable of engendering meaningful differences in cranial shape.

In characterising Jomon diet based on the archaeological record, as we have done here, we are relying on an imperfect proxy; more accurate reconstructions of dietary differences within and between regions may be achieved by direct analyses such as isotope analyses of skeletal remains. To date, most isotope studies of Jomon diet have tended to focus on a single or few similar sites rather than regional comparisons (e.g., Naito et al. 2010, 2013; Kanazawa-Kiriyama and Kusaka 2017; Kusaka 2019), partly due to complications such as marine reservoir and local temperature effects (Yoneda et al. 2004; Chisholm 2004; Naito et al. 2010). The limited evidence available shows nitrogen isotopes from skeletons from Kitokogane (Hokkaido) resemble those of large fish and sea mammals, whilst those of Jomon from inland central Honshu, such as Kitamura (Nagano), and South/West Japan regions, such as Yosekura (Chugoku), appear more like large herbivores in (Akazawa and Maeyama 1986). Indeed, there seems to be a decreasing cline in dietary marine protein from Hokkaido, through central Honshu to western Japan (Chisholm 2004), although the source of protein does not speak to its sufficiency or otherwise. A more recent study (Kusaka et al. 2010) compared several sites in the Sanyo (South/West Japan region in this study) and Tokai (coastal central Honshu region in this study) regions. Kusaka et al. found isotopic signatures concurrent with a mixture of marine and terrestrial proteins in both regions, but variation within and between sites and regions. Although the sites within Sanyo varied, there were actually higher nitrogen signals from this region compared to the Tokai individuals. The authors suggest this might be due to a greater reliance on shellfish in Tokai, a region famous for its middens, and more high trophic level marine fish in Sanyo (Kusaka et al. 2010). These studies suggest there may be meaningful differences in protein intake between regions, but that the storey is complex and needs further investigation, preferably using isotopic methods that allow the interpretation of the whole diet, rather than just its protein portion (Kusaka 2019). Archaeobotanical analyses of food remain in dental calculus (e.g., Salazar-García et al. 2013; Henry et al. 2014) and residue on cooking/food preparation items (e.g., Junno et al. 2020; Robson et al. 2020; Saul et al. 2012; Yasui 2022) could also improve the resolution of our understanding.

### Variation in Jomon diet within regions

Our results suggest that regional dietary differences may contribute to the neurocranial (and particularly temporalis region) shape variation amongst the Jomon, yet this is not a strong signal. In addition to the potential non-dietary factors acting on Jomon cranial shape, one of the reasons for the signal’s weakness may be the scale of variation in Jomon diets at many different levels: seasonally, between different groups of people within a site, over time, etc. During the Jomon period, the Japanese Archipelago was an extremely rich environment for foragers and all Jomon diets appear to have encompassed great variation, both within and between seasons (Aikens and Higuchi 1982; Kobayashi et al. 2004). The archaeological record shows Jomon exploitation of > 60 mammal species, > 350 species of shellfish, 70 species of fish, and 35 species of bird; at least 55 species of plants have also been found, but this likely samples a fraction of what was exploited, due to preservation bias (Kobayashi et al. 2004). Although not all species represented were necessarily food, this remains evidence of a very varied diet. It is possible that in this situation, dietary variation between sites is not large enough to affect the cranium sufficiently for the method used here to capture it completely, hampered as we were by small sample sizes and fragmentary remains, or that only subtle variation in morphology exists due to the extent of intraregional dietary variation. Most investigations of the dietary influences on craniofacial morphology have considered differences between the markedly divergent subsistence strategies of foragers and farmers (Menéndez and Buck 2022), although see for example, (Hoover and Williams 2016; Noback and Harvati 2015; Katz et al. 2017). Hoover and Williams (2016) did identify mandibular differences between dietary groups of Jomon, as discussed above, but crania are likely less plastic than mandibles in response to dietary strains (von Cramon-Taubadel 2011). This may be expected due to the more numerous competing selection pressures acting on the cranium from the need to protect and house the sense organs, mastication, speech, and so on. This interplay of competing requirements is hypothesised to lead to high levels of canalisation (Buck et al. 2010; Stock and Buck 2010).

Some isotope analyses of Jomon diet have suggested sex differences in diet within single sites. For example, at the Ota site in the Sanyo region of southwest Honshu, males seem to have had a greater marine component to their diets and females a greater reliance on terrestrial foods (Kusaka et al. 2010). Kusaka et al. (2010) suggest this is likely due to sexual division of subsistence practises, a plausible suggestion given that extant hunter-gatherers are known to consume a considerable proportion of their daily food intake whilst foraging for it (Berbesque et al. 2016). Sex differences in diet do not seem to have been a culture-wide division however, as no sex differences in isotopic signature have been found at sites such as Boji in the central highlands of Honshu (Yoneda et al. 2004). Identification and interpretation of these subtle inter-group differences depends on comprehensive chemical analysis of skeletal remains using both carbon and nitrogen isotopes and considering the signature of both bone collagen and hydroxyapatite (Kusaka 2019). At Yoshigo, on the East coast of central Honshu, there is evidence for sex differences in protein (Kusaka et al. 2010) but these are obscured when the diet is analysed as a whole (carbohydrates, lipids, and proteins) (Kusaka 2019). Although we found no effect of sexual dimorphism in this study, our samples for each population were small and some necessarily had unbalanced sex ratios. It is thus possible that differences in diet between the sexes is a factor obscuring larger scale, regional dietary variation.

The Jomon era is divided into chronological periods (Incipient to Epi-Jomon) and cultural and subsistence practises, together with demography and skeletal morphology, changed over time as well as geography (Koyama 1978; Yasuda 1978; Teruya 1986; Temple 2008; Kaifu 2010; Crema et al. 2016). For reasons of sample size optimisation, our study groups together individuals from ~ 6,100–2,300 BP, encompassing the Early to Final Jomon periods (see details in Buck et al. 2019). Combining time periods within groups could conceal or dilute morphological patterns related to diet and/or changes in diet through time, as we earlier suggested could contribute to the lack of an ecogeographic signal in our sample (Buck et al. 2019). Temple has suggested that increasing population density over time in western Honshu led to a decrease in stature in the Late/Final Jomon, due to food competition, malnutrition, and chronic infection (Temple 2008). Concomitantly, an increase in caries between Middle and Late/Final Jomon in both East and West Japan suggests a change in diet within regions, perhaps due to climate cooling and probably leading to a greater proportion of plant food (Temple 2007), a suggestion supported by the isotope values from sites such as Yoshigo, which show less reliance on marine food sources over time (Kanazawa-Kiriyama and Kusaka 2017). It is also towards the end of the Jomon period that cultivation of some plant foods, such as buckwheat and millet, begins in earnest (Habu 2004; Kobayashi et al. 2004), fundamentally changing the Jomon diet and way of life. In addition to the direct effects of climate change on resource availability, sea-level rise over the Jomon period following the warming after the last glacial maximum would also have re-shaped the landscape and Jomon subsistence and demography over the period in question (Koyama 1978; Yasuda 1978; Akazawa 1986a). These factors and many others will have interacted, with the result that the diets of individuals from the same dietary region, but different time periods, will have experienced different dietary selection pressures on their crania. This may explain why Jomon crania do not reflect regional diets as clearly as mandibles (but see Kaifu 2010) or material culture.

### Signatures of population history

There are no patterns resembling those resulting from isolation by distance in any of the analyses of cranial size or shape. This suggests that the stochastic accumulation of neutral variation across geographic space is not a major influence on cranial form (shape and size) amongst the Jomon studied here. In these analyses, we have only examined the first two principal components of shape, it is possible that patterns of population history would be found on higher PCs, and that this factor does contribute to variation between sites, but it is not one of the main factors determining that variation. In contrast to the global scale, where neutral processes are thought to be the strongest influence on cranial form (Roseman 2004; Roseman and Weaver 2004; Betti et al. 2009, 2010), our results suggest that at a regional level (at least in this time and place) other factors are prevalent.

The lack of a pattern that resembles population history results from some relatively distant groups being more similar to one another than would be expected under an isolation by distance model, and simultaneously other geographically close groups being morphologically more different than would be expected. Comparing our results to those of previous authors who have conducted interregional analyses of Jomon cranial morphology, or comparing amongst those previous analyses (e.g., Matsumura 1989, 2007; Kondo 1994; Adachi et al. 2021), is not always straightforward for methodological reasons. ‘Region’ is an imprecise term that is not always defined, and the sites and individuals included in each study differ. This has led to a situation where studies appear to contradict one another but may actually be analysing different things. Here, we chose to investigate the relationship between geography and morphology at a site level as the closest possible approximation of population-level variation. The low levels of variation we see here between some widely-spaced sites are in keeping with previous reports of morphological homogeneity between sites (Matsumura 1989, 2007; Hanihara 1991), yet we also see high levels of differentiation for some greater distance classes. This disparity could be due to complex patterns of residence and kinship amongst the studied populations resulting in differences in gene flow across regions. Where we see high levels of intra-site variation relative to between-site variation, this echoes the findings of Kondo (1994), who found high levels of inter-site variation in males within a region compared to sites in different regions. For example, individuals from different sites in northern Chiba were as morphologically distant from one another as they were from sites in the Atsumi Peninsula (Kondo 1994). This picture of random patterning and of both continuity and heterogeneity is supported by the complexity of the genetic evidence. A recent analysis shows similar Y-chromosome haplogroup frequencies between Higashimyo (Kyushu, South/West Japan) and Funadomari (Hokkaido) individuals (Adachi et al. 2021), but also suggests rapid diffusion and differentiation of Jomon groups throughout the Archipelago, followed by low levels of subsequent of regional interactions and migrations (Adachi et al. 2021). Ancient DNA studies to date, based mainly on mtDNA and Y chromosome data, show a multifaceted story of migration, genetic drift, and isolation, but no marked archipelago-wide patterns of differentiation (Adachi et al. 2013, 2021; Kanzawa-Kiriyama et al. 2017; Mizuno et al. 2023).

Greater than expected differentiation between geographically close groups could have resulted from social factors structuring variation at a local scale. Cultural divisions such as those suggested by regional styles of pottery (Aikens and Higuchi 1982; Akazawa 1988; Kobayashi et al. 2004) and patterns of dental ablation and modification (Harunari 1986) could have resulted in barriers to inter-marrying between certain groups, complicating a straightforward isolation by distance pattern. In contrast, greater than expected similarity in cranial shape between some of the more distant groups in the current sample could result from gene flow between these groups, perhaps facilitated by trade networks (Kobayashi et al. 2004). These networks appear in the archaeological record as the presence of high-value goods, such as jadeite, at long distances from their origin, and in evidence of a continuous, overarching Jomon culture throughout Japan, particularly in the ceramic tradition (Teruya 1986; Kobayashi et al. 2004). The complex geography of the Japanese Archipelago is another potential factor leading to a lack of spatial autocorrelation in Jomon cranial shape amongst populations that are close to each other. Multiple islands and mountains of considerable height would have impeded land travel; travel around the Japanese Archipelago before roads and railways would often have been simpler by water than by land, meaning that groups close to one another ‘as the crow flies’ did not actually exchange genes more regularly than groups separated by greater but more easily traversable distances (Kobayashi, et al. 2004).

### Alternative influences on cranial morphology

In this paper and in our previous work (Buck et al. 2019), we have examined the effects of some of the most fundamental influences on human cranial morphology: climate, diet, and population history, yet this list is far from exhaustive. A further example would be human cultural practises that actively change the morphology of the cranium. As discussed above, the ritual removal of healthy adult teeth, ablation, was common in many Jomon populations including at the Yoshigo, Ebishima, and Tsukumo sites included in the current study, where 80–100% of all adults had at least one tooth removed (Harunari 1986; Mizoguchi and Dodo 2001). Dental modification is not exclusive to the Jomon and has been found in groups widely separated in time and space, from Epipalaeolithic North African Iberomaurusian culture (De Groote and Humphrey 2016) to Neolithic Italy (Robb 1997). Analysis of the Iberomaurusian evidence suggests that the changes made by ablation to the biomechanical strains produced by chewing affect craniofacial morphology, at least in the lower face (Balzeau and Badawi-Fayad 2005). Thus, some effect of this practise on the craniofacial shape of the Jomon could have confounded adaptation to their diet.

## Conclusions

In this study, and our previous work, we have compared how climate, diet, and population history covary with, and may influence, cranial shape in an ecologically widespread prehistoric forager culture. In contrast to non-human primates inhabiting the substantially similar environments, Jomon foragers show no relationship between climate or population history and cranial morphology. There are, however, relatively subtle differences in overall neurocranial shape, and in the region of the temporalis muscle, between groups with archaeologically inferred dietary differences. These findings could be attributed to differences in biomechanical regimes related to those diets. The relationships between diet, environment, and culture are extremely difficult to unpick, however. More work is needed to directly analyse different diets within and between groups and the nutritional and biomechanical properties of the foods making up those diets (e.g., protein content and hardness). Our results demonstrate the intricacies of dietary variation and caution against solely relying on broad-brushstroke comparisons such as forager/farmer. They also emphasise the importance of the interrelationship between culture and environment in human adaptation and underline the multiplicity of interrelated variables acting on human skeletal morphology, which differs at the species, regional, population, and individual level.

### Supplementary Information

Below is the link to the electronic supplementary material.

Supplementary file1 (PDF 744 KB)
