# Supplementary material for: Factors influencing cranial variation between prehistoric Japanese forager populations
Source: Archaeol Anthropol Sci. 2023 Dec 13;16(1):3. doi: 10.1007/s12520-023-01901-6 (PMC10716076; doi:10.1007/s12520-023-01901-6)
Supplement: Supplementary file 1 — Supplementary file1 (PDF 744 KB) [file 12520_2023_1901_MOESM1_ESM.pdf]

Buck et al. Supplemental information

1. Detailed explanation of dietary classifications
2. Table showing details of sample for each analysis
3. Landmark definitions for each landmark set
4. Wireframes for each landmark set
5. Results excluding inland coastal Honshu group
6. Size and sexual dimorphism results
7. Bibliography

1. Detail on dietary grouping in this study

The Jomon archaeological record is extremely rich and well-studied, allowing detailed reconstruction of subsistence practices and diets. Here we employ a geographical dietary classification based on the pioneering work of Akazawa (Akazawa, 1982, 1986), but we also include modifications suggested by a more recent, geometric morphometric methods-based study by Hoover and Williams (2015). The most fundamental division in Akazawa's scheme is an East / West split throughout the length of the Japanese Archipelago (Figure S1). This division corresponds to an important ecological divide that affects much of Japan's flora and fauna (Yasuda, 1978; Akazawa, 1982; Tsukada, 1986) and its effect on Jomon diet, as recognised by Akazawa, has been borne out by subsequent researchers (Kobayashi, 2004; Temple, 2007). Akazawa subsequently subdivides his Eastern classification into three regions from North to South: costal Tohoku / Hokkaido, lowland coastal East Kanto / Tokai, and the area around the Sea of Japan. The coastal regions of Tohoku and Hokkaido have the greatest density of Jomon-era shell middens, illustrating the importance of shellfish in this region. At these midden sites, associated archaeology shows hunting of marine mammals and migratory fish. Sites in costal Kanto and Tokai in Eastern Japan yield a different technology demonstrating a reliance on estuarine and marine fish. The most characteristic tools from more southerly Sea of Japan area in Eastern Japan are projectile points, suitable for the hunting of terrestrial game. In contrast, Western Japanese Jomon material culture shows the exploitation of freshwater fish and technological evidence of greater harvesting and processing of vegetable foods. Hoover and Williams' (2015) used a very similar scheme of regional dietary variation to Akazawa's to demonstrate a relationship between diet and mandibular morphology Jomon remains. The main difference between Akazawa's and Hoover and Williams' dietary regions is that the latter separated out sites in Hokkaido from Northeastern Honshu, due to the far greater reliance on marine mammals in Hokkaido (Oxenham and Matsumura, 2008).

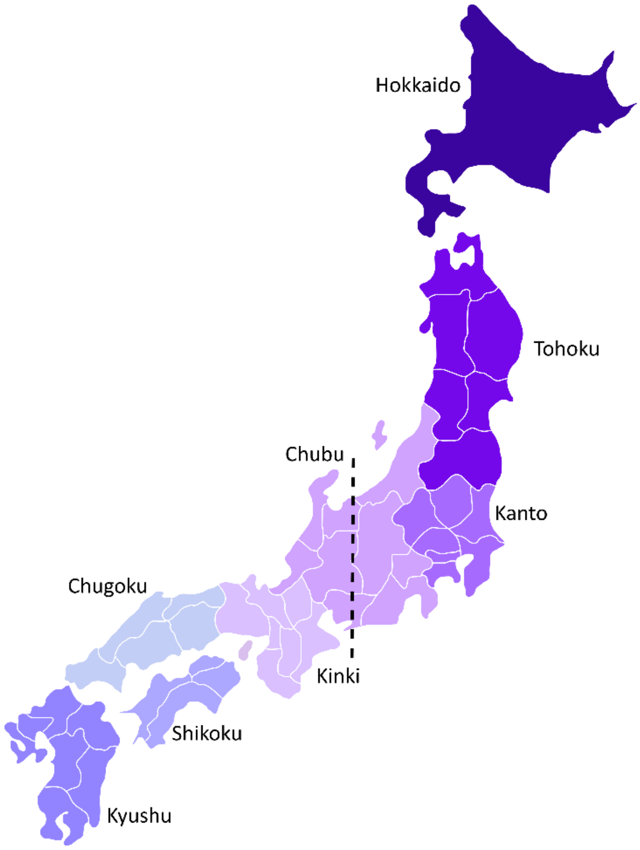

Figure S1: Map of Japan showing the main regions of Japan as referred to in the text discussing Akazawa's work (see above). The dashed line is the approximate location of the major East / West division in ecological zones referred to in Akazawa (Akazawa, 1982) as falling at 137° NE. For dietary groupings, see Figure 1, main text.

In our combined scheme (see Figure 1, main text), we classify our samples into the following dietary groups: Hokkaido, northeastern Honshu, coastal central Honshu, inland central Honshu, and South / West Japan (Table 1, Figure 1). The material culture from each region suggests the following dietary characteristics: Hokkaido diet has a high marine component, with fishing of rocky coast and migratory fish and a substantial, specialised marine mammal component. The diet of the Jomon from the rocky coast of northeastern Honshu also shows heavy reliance on shellfish and migratory fish, but lacks the reliance on marine mammals seen in Hokkaido. Diet in coastal central Honshu area, covering the Kanto coastal lowland region around Tokyo is characterised by riverine, estuarine, and sheltered coastal resources. In the inland central Honshu area, diet includes plant and freshwater resources but differs from other regions by showing greater importance of terrestrial hunting. In South / West Japan the archaeology shows the exploitation of freshwater fish and a more substantial dependence on vegetable foods.

2. Sample details

Table S1: Sample for each analysis, showing site and sex.

**Craniofacial / facial landmark set**				**Neurocranial landmark set**				**Temporalis landmark set**				**Bite force landmark set**			
**Site**	**Sex**	**n**	**Site total**	**Site**	**Sex**	**n**	**Site total**	**Site**	**Sex**	**n**	**Site total**	**Site**	**Sex**	**n**	**Site total**
Funadomari	F	1		Funadomari	F	2		Funadomari	F	2		Funadomari	F	2	
	M	1			M	2			M	2			M	1	
	U	0	2		U	1	5		U	0	4		U	1	4
Kitakogane	F	0		Irie	F	0		Irie	F	0		Irie	F	0	
	M	2			M	1			M	1			M	1	
	U	0	2		U	1	2		U	1	2		U	0	1
Kotan Onsen	F	1		Kitakogane	F	1		Kitakogane	F	1		Kitakogane	F	1	
	M	1			M	2			M	1			M	2	
	U	0	2		U	1	4		U	0	2		U	0	3
Takasago	F	1		Kotan Onsen	F	1		Kotan Onsen	F	1		Kotan Onsen	F	1	
	M	1			M	2			M	2			M	1	
	U	1	3		U	0	3		U	1	4		U	1	3
Ebishima	F	1		Takasago	F	1		Takasago	F	0		Takasago	F	0	
	M	4			M	2			M	1			M	2	
	U	0	5		U	1	4		U	1	2		U	1	3
Miyano	F	0		Ebishima	F	9		Ebishima	F	8		Ebishima	F	3	
	M	2			M	8			M	4			M	4	
	U	0	2		U	0	17		U	0	12		U	0	7
Yoshigo	F	1		Miyano	F	0		Miyano	F	0		Miyano	F	0	
	M	1			M	3			M	3			M	3	
	U	0	2		U	0	3		U	0	3		U	0	3
Wakaumi	F	0		Yoshigo	F	6		Yoshigo	F	3		Yoshigo	F	0	
	M	1			M	5			M	3			M	2	
	U	0	1		U	0	11		U	0	6		U	0	2

Tochibara	F	1	
	M	0	
	U	0	1
Ikawazu	F	0	
	M	1	
	U	0	1
Tuskumo	F	5	
	M	4	
	U	0	9
Goryo	F	0	
	M	1	
	U	0	1
Yamaga	F	2	
	M	0	
	U	0	2
**Total**		**33**	

Wakaumi	F	0	
	M	1	
	U	0	1
Tochibara	F	3	
	M	0	
	U	0	3
Ikawazu	F	1	
	M	2	
	U	1	4
Tuskumo	F	10	
	M	9	
	U	1	20
Einomaru	F	1	
	M	0	
	U	0	1
Goryo	F	0	
	M	1	
	U	0	1
Todoroki	F	1	
	M	0	
	U	0	1
Yamaga	F	2	
	M	1	
	U	0	3
**Total**			**83**

Wakaumi	F	0	
	M	1	
	U	0	1
Tochibara	F	2	
	M	0	
	U	0	2
Ikawazu	F	1	
	M	2	
	U	0	3
Tuskumo	F	6	
	M	6	
	U	0	12
Einomaru	F	1	
	M	0	
	U	0	1
Goryo	F	0	
	M	1	
	U	0	1
Todoroki	F	0	
	M	0	
	U	1	1
Yamaga	F	2	
	M	2	
	U	0	4
**Total**			**60**

Wakaumi	F	0	
	M	1	
	U	0	1
Tochibara	F	1	
	M	0	
	U	0	1
Ikawazu	F	1	
	M	2	
	U	0	3
Tsukumo	F	6	
	M	6	
	U	0	12
Einomaru	F	1	
	M	0	
	U	0	1
Goryo	F	0	
	M	1	
	U	0	1
Yamaga	F	1	
	M	1	
	U	0	2
**Total**			**47**

3. **Landmark definitions for each landmark set.**

Table S2: Craniofacial landmark set of 37 landmarks. Inf: inferior, ant: anterior, sup: superior, pos: posterior, see Figure 2, main text.

Name	Number in craniofacial landmark set
Glabella	1
Nasion	2
Supraorbital notch	3
Mid-torus inf.	4
Frontomalare orbital	5
Zygoorbitale	6
Zygomaxillare	7
Alare	8
Nasiospinale	9
Prosthion	10
Prosthion 2	11
Dacryon	12
Frontomalare temporale	13
Zygomatic arch / alisphenoid / frontal	14
Pterion pos.	15
Zygomatic arch ant.	16
Zygotemporale sup.	17
Zygotemporale inf.	18
Porion	19
Asterion	20
Inion	21
Lambda	22
Bregma	23
Frontotemporale	24
Zygomatic process pos.	25
Opisthion	26
Basion	27
Articular tubercule	28
Post-glenoid process dist.	29
Temporal zygomatic curve pos.	30
Petrous / alisphenoid / zygomatic	31
Maxilla / palate	32
Incisivion	33
P3 / 4	34
P4 / M1	35
M1 / 2	36
M2 / 3	37

Table S3 Facial landmark set, 22 landmarks. Inf: inferior, ant: anterior, sup: superior. See Figure 2, main text.

Name	Number in facial landmark set
Glabella	1
Nasion	2
Supraorbital notch	3
Mid-torus inf.	4
Frontomalare orbital	5
Zygoorbitale	6
Zygomaxillare	7
Alare	8
Nasiospinale	9
Prosthion	10
Prosthion 2	11
Dacryon	12
Frontomalare temporale	13
Zygomatic arch ant.	14
Zygotemporale sup.	15
Zygotemporale inf.	16
Maxilla / palate	17
Incisivion	18
P3 / 4	19
P4 / M1	20
M1 / 2	21
M2 / 3	22

Table S4: Neurocranial landmark set, 9 landmarks. Pos: posterior. See Figure 2, main text.

Name	Number in neurocranial landmark set
Glabella	1
Pterion pos.	2
Porion	3
Asterion	4
Inion	5
Lambda	6
Bregma	7
Frontotemporale	8
Zygomatic process pos.	9

Table S5: Temporalis landmark set, 6 landmarks (Paschetta et al., 2010). Pos: posterior. See Figure 2, main text.

Name	Number in neurocranial landmark set
Zygomaxillare	1
Stephanion	2
Ethomion	3
Pos. infratemporal fossa	4
MW1	5
MW2	6

Table S6: Bite force landmarks and semilandmarks. See Figure 2, main text.

Name	Number in bite force landmark set	Type of landmark
M2	1	True landmark
Frontomalare temporale	2	True landmark
Temporomalare sup	3	True landmark
Temporomalare inf	4	True landmark
Temporomalare inf int	5	True landmark
Zygomaxillare pos	6	True landmark
TMJ	7	True landmark
Temporosphenoidale	8	True landmark
Frontotemporale	9	True landmark
Stephanion	10	True landmark
Auriculare (porion)	11	True landmark
Asterion	12	True landmark
Inf malar 1	13	Semilandmark curve 1
Inf malar 2	14	Semilandmark curve 1
Inf malar 3	15	Semilandmark curve 1
Sup malar 1	16	Semilandmark curve 2
Sup malar 2	17	Semilandmark curve 1
Sup malar 3	18	Semilandmark curve 1
Sup malar 4	19	Semilandmark curve 1
Mid curve 1	20	Semilandmark curve 3
Mid curve2	21	Semilandmark curve 3
Mid curve 3	22	Semilandmark curve 3
Mid curve 4	23	Semilandmark curve 3
Mid curve 5	24	Semilandmark curve 3

Mid curve 6	25	Semilandmark curve 3
Mid curve 7	26	Semilandmark curve 3

4. Wireframes for each landmark set

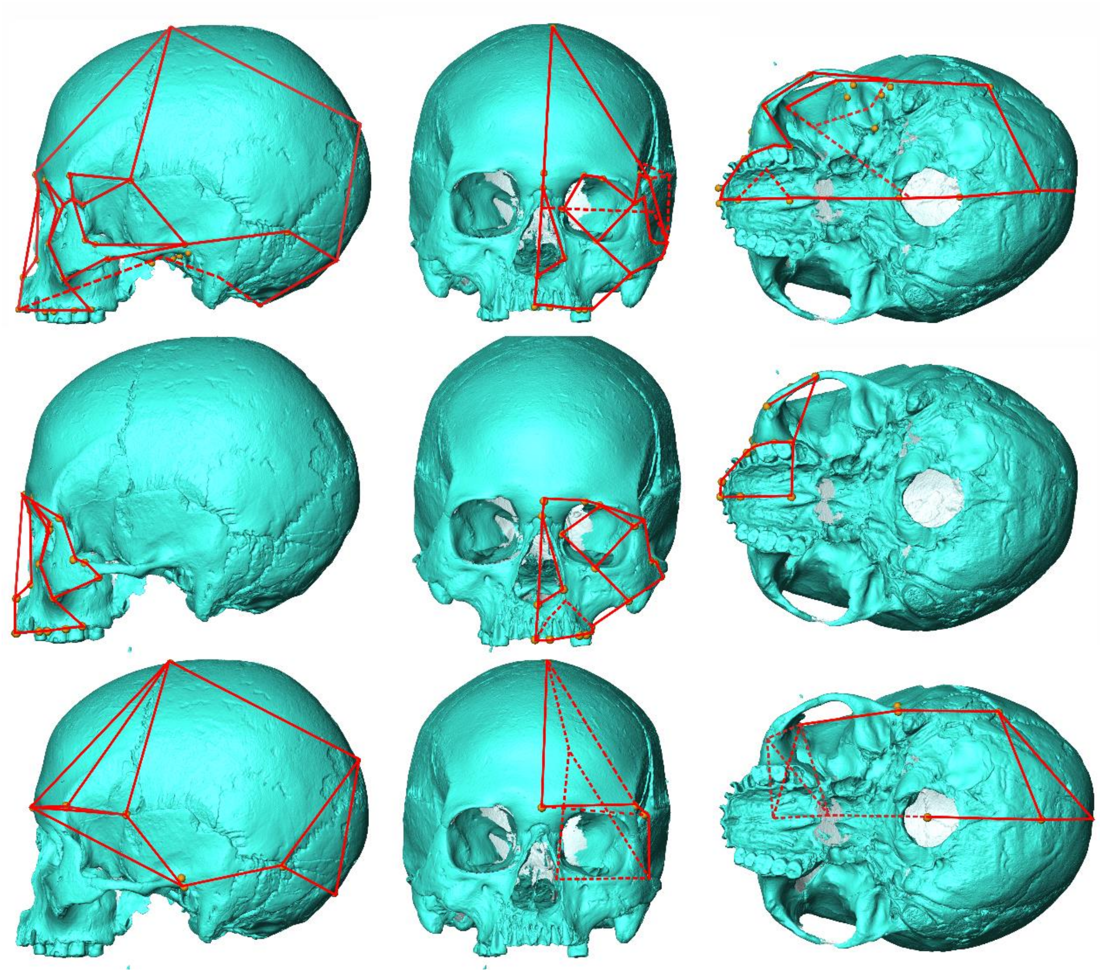

Figure S2: Wireframes joining landmarks in craniofacial (top), facial (middle) and neurocranial (bottom) landmark sets from lateral, frontal and basicranial views. These wireframes are used in subsequent figures to facilitate visualisation.

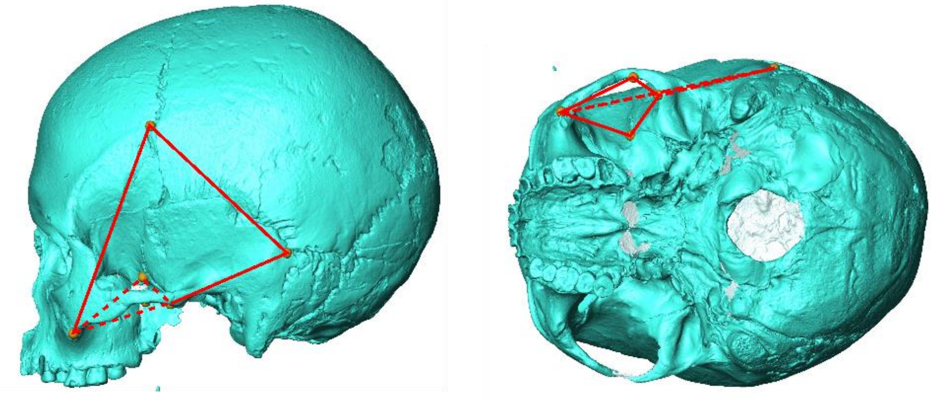

Figure S3: Wireframes joining landmarks in temporalis region landmark set. These wireframes are used in subsequent figures to facilitate visualisation.

5. Results excluding inland coastal Honshu group

Craniofacial

MANOVA: Differences between groups not significant ($F(3,28) = 1.06$, $p > 0.05$), as when ICH is included ($F(4,28) = 1.09$, $p > 0.05$).

Facial

MANOVA: Differences between groups not significant ($F(3,28) = 1.35$, $p > 0.05$), as when ICH is included ($F(4,28) = 1.22$, $p > 0.05$).

Neurocranial

MANOVA: Significant difference between dietary groups when ICH is excluded ($F(3,76) = 1.64$, $p < 0.01$), as when it is included ($F(4,78) = 1.60$, $p < 0.005$).

Temporalis:

MANOVA: Significant difference between dietary groups when ICH is excluded ($F(3,55) = 1.68$, $p < 0.05$). This remains unchanged from the result with ICH included ($F(4,56)= 2.30$, $p < 0.001$).

Bite force:

No difference in ANOVA results because not included in bite force ANOVA because >1 individual is required in each group.

6. Size and sexual dimorphism results

Scatterplots for two variables (craniofacial shape and temporalis shape) that showed significant relationships with size (see main text).

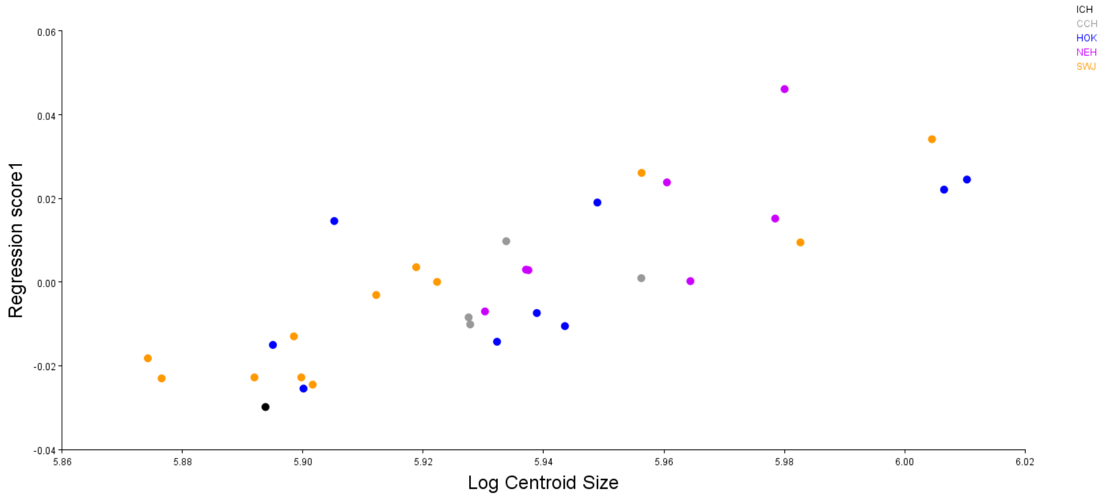

Figure S4: Craniofacial shape against Ln centroid size. Coloured by diet group, Hokkaido: dark blue, northeastern Honshu: magenta, inland central Honshu: black, coastal central Honshu: grey, South / West Japan: orange.

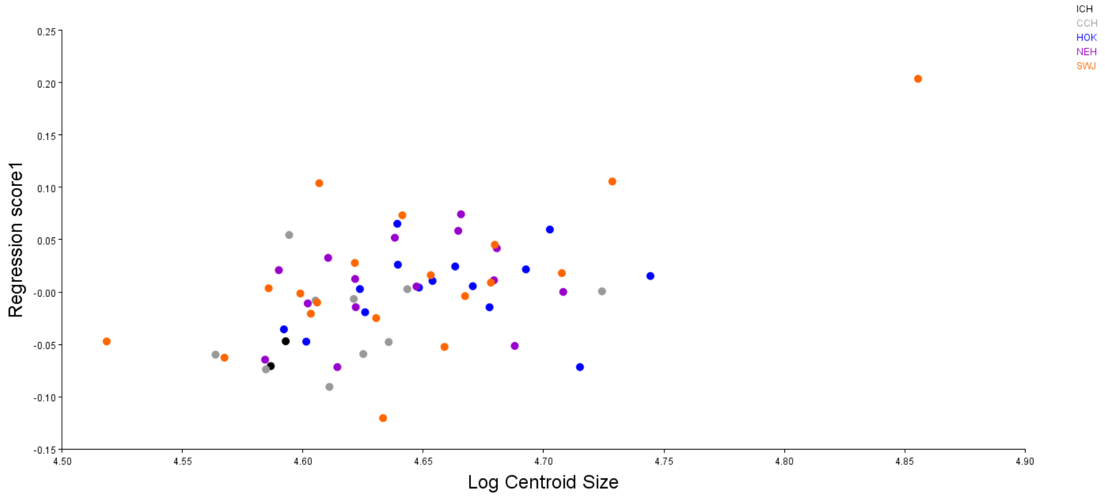

Figure S5: Temporalis shape against Ln centroid size. Coloured by diet group, Hokkaido: dark blue, northeastern Honshu: magenta, inland central Honshu: black, coastal central Honshu: grey, South / West Japan: orange.

There is a significant relationship between the size and dietary group in both the craniofacial and temporalis region analyses. Note that as explained in the main text, this is the natural log of the centroid size of each landmark set, thus in the case of the temporalis region it is not relative to the overall size of the cranium. In each case, size only explains a relatively small (<5%) amount of variation in the shape of the cranial region in question and there seems to be no clear difference in this relationship among dietary groups (see Figures S4 and S5). This suggests that diet-related allometry is not a major factor in shaping cranial variation among this sample of Jomon.

Bite force and sexual dimorphism

If bite force in males and females (i.e., those individuals classified as 'uncertain' are removed, leaving a sample of $n = 40$) is adjusted for sex using the formula in Ackermann et al. (Ackermann et al., 2006), there is still no significant difference in bite force between dietary groups ($F(3,37) = 0.662$, $p > 0.05$).

7. Bibliography

Akazawa, T., 1982. Jomon people subsistence and settlements: discriminatory analysis of the Later Jomon settlements.

Akazawa, T., 1986. Huntergatherer adaptations and the transition to food production in Japan. In: Zvelebil, M. (Ed.), Hunters in Transition: Mesolithic Societies of Temperate Eurasia and Their Transition to Farmining. Cambridge University Press, Cambridge, pp. 151–166.

Hoover, K.C., Williams, F.L., 2015. Variation in regional diet and mandibular morphology in prehistoric Japanese hunter–gatherer–fishers. Quaternary International. 1–9.

Kobayashi, T., 2004. Jomon reflections: forager life and culture in the prehistoric Japanese Archipelago. Oxbow Books, Oxford.

Oxenham, M.F., Matsumura, H., 2008. Oral and Physiological Paleohealth in Cold Adapted Peoples: Northeast Asia, Hokkaido. American Journal of Physical Anthropology. 135, 64–74.

Paschetta, C., de Azevedo, S., Castillo, L., Martínez-Abadías, N., Hernández, M., Lieberman, D.E., González-José, R., 2010. The influence of masticatory loading on craniofacial morphology: A test case across technological transitions in the Ohio valley. American journal of physical anthropology. 141, 297–314.

Temple, D.H., 2007. Dietary variation and stress among preshistoric Jomon foragers from Japan. 133, 1035–1046.

Tsukada, M., 1986. Vegetation in prehistoric Japan: the last 20,000 years. In: Pearson, R.J., Barnes, G.L., Hutterer, K.L. (Eds.), Windows on the Japanese Past: Studies in Archaeology and Prehistory. : Center for Japanese Studies, University of Michigan, Ann Arbor, pp. 11–56.

Yasuda, Y., 1978. Prehistoric environment in Japan: Palynological approach. Institute of Geography: Tohoku University, Sendai.
